# Tumor infiltration of inactive CD8 + T cells was associated with poor prognosis in Gastric Cancer

**DOI:** 10.1007/s10120-024-01577-4

**Published:** 2024-12-25

**Authors:** Naoki Katayama, Kenoki Ohuchida, Kiwa Son, Chikanori Tsutsumi, Yuki Mochida, Shoko Noguchi, Chika Iwamoto, Nobuhiro Torata, Kohei Horioka, Koji Shindo, Yusuke Mizuuchi, Naoki Ikenaga, Kohei Nakata, Yoshinao Oda, Masafumi Nakamura

**Affiliations:** 1 Department of Surgery and Oncology, Graduate School of Medical Sciences, Kyushu University, 3-1-1 Maidashi, Higashi-Ku, Fukuoka, 812-8582 Japan; 2 Department of Advanced Medical Initiatives, Graduate School of Medical Sciences, Kyushu University, Fukuoka, Japan; 3 Department of Anatomic Pathology, Graduate School of Medical Sciences, Kyushu University, Fukuoka, Japan

**Keywords:** Gastric cancer, CD8-positive T-cells, Tumor-infiltrating lymphocytes, Interferon gamma, Immunosuppressive cell

## Abstract

**Background:**

Gastric cancer (GC) shows limited response to immune checkpoint inhibitors due to its complex tumor immune microenvironment (TIME). This study explores the functions of various immune cells in the complex TIME in GC.

**Methods:**

We assessed CD8 + T-cell infiltration of GC tissues by immunohistochemistry, and performed single-cell RNA sequencing (scRNA-seq) of tumor and normal tissues from 34 patients with GC.

**Results:**

We categorized 157 GC patients into LOW, MID, and HIGH groups based on their CD8 + T-cell infiltration. Overall survival was notably lower for the HIGH and LOW groups compared with the MID group. Our scRNA-seq data analysis showed that CD8 + T-cell activity markers in the HIGH group were expressed at lower levels than in normal tissue, but the T-cell-attracting chemokine CCL5 was expressed at a higher level. Notably, CD8 + T-cells in the HIGH group displayed lower PD1 expression and higher CTLA4 expression. TCR repertoire analysis using only Epstein–Barr virus-negative cases showed that CD8 + T-cell receptor clonality was lower in the HIGH group than in the MID group. Furthermore, in the HIGH group, the antigen-presenting capacity of type 1 conventional dendritic cells was lower, the immunosuppressive capacity of myeloid-derived suppressor cells was higher, and the expression of CTLA4 in regulatory T-cells was higher.

**Conclusion:**

The present data suggest that the infiltration of inactive CD8 + T-cells with low clonality is induced by chemotaxis in the HIGH group, possibly leading to a poor prognosis for patients with GC.

**Supplementary Information:**

The online version contains supplementary material available at 10.1007/s10120-024-01577-4.

## Introduction

Gastric cancer (GC) is a major global cancer, accounting for 1 in 13 cancer deaths worldwide in 2020, ranking fifth in incidence and fourth in mortality [1]. East Asia has the highest incidence, with a 5-year survival rate of 7% for patients with GC with distant metastasis [2]. The rise of immune checkpoint inhibitors has advanced tumor immune microenvironment (TIME) analysis, especially of T-cells. PD1 and CTLA4 are key immune checkpoint molecules on T-cells, suppressing T-cell activity by binding to specific ligands [3, 4]. Tumors with high immune infiltration levels are considered sensitive to immune checkpoint inhibitors, but inhibitors have limited efficacy in GC [5]. Therefore, an in-depth analysis of the complex TIME is needed.

In GC, CD8 + tumor-infiltrating lymphocyte (TIL) abundance has been reported to correlate with better postoperative clinical outcomes and is an independent prognostic factor for overall survival by multivariate analysis [6]. In addition, CD8 + TILs express high levels of immune checkpoint molecules, such as PD1, which contribute to antitumor immunosuppression [7, 8]. However, several recent studies involving genetic analysis have reported that abundant CD8 + T-cell infiltration in a specific subset of GC microenvironments is associated with low expression of immune checkpoint molecules and poor prognosis [9, 10]. These findings suggest that immunological heterogeneity and complex immunosuppression occur within the CD8 + TIL-rich tumor environment, but details of immune cell functional status and the mechanism of action remain unknown.

In this study, we classified patients with GC based on the ratio of CD8 + TILs to cancer cells to elucidate the function of various immune cells in diverse TIMEs.

## Materials and methods

### Human GC sample collection

We performed single-cell RNA sequencing (scRNA-seq) using samples from 34 patients with GC that were diagnosed clinically and pathologically at Kyushu University Hospital. Clinical data, including histopathological information, were collected from electronic medical records. See the supplementary materials for details.

### scRNA-seq and single-cell T-cell receptor sequencing data processing

scRNA-seq and single-cell T-cell receptor (TCR) sequencing data were aligned to the human genome (GRCh38) using the 10 × Genomics Cell Ranger pipeline (v5.0.0) to generate gene-barcode matrices, which were processed with the Seurat R package (v4.2.1). See the supplementary materials for detailed preparation and analysis information.

### Immunohistochemistry and immunofluorescence

Immunohistochemistry and immunofluorescence were performed on samples from patients who underwent GC surgery and histopathologic diagnosis at Kyushu University Hospital. We analyzed 157 tumor samples. See the supplementary materials for detailed information.

### Statistical methods

Statistical analyses, except for scRNA-seq analyses, were performed using GraphPad Prism 10 (v10.2.1). Survival analysis was performed using the Cox proportional hazards model. Survival curves (recurrence-free survival [RFS] and overall survival [OS]) were generated with the Kaplan–Meier method and compared using the log-rank test. Chi-squared tests were used to evaluate correlations between clinicopathological characteristics and groups based on ranges of CD8 + TILs (Table S1). The Wilcoxon rank-sum and Kruskal–Wallis tests were used for scRNA-seq data. Statistical significance was set at *p* < 0.05 (ns: *p* ≥ 0.05, ∗ : *p* < 0.05, ∗∗ : *p* < 0.01, ∗∗∗ : *p* < 0.001, and ∗∗∗∗: *p*< 0.0001).

## Results

### Prognostic impact of CD8 + T-cell infiltration

**Fig. 1:**
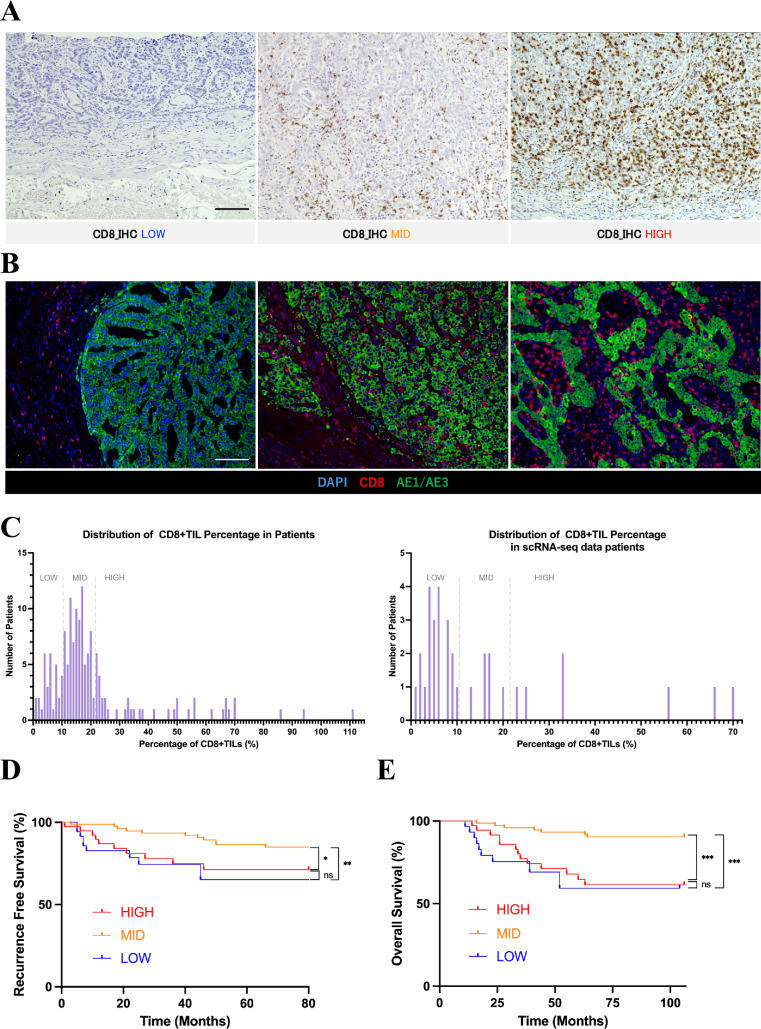
Prognostic impact of the degree of CD8 + T-cell infiltration. **A** Representative immunohistochemistry (IHC) images of CD8 immunostaining in gastric cancer (GC) and adjacent tissues from CD8_IHC LOW, MID, and HIGH groups (from left to right). Scale bars, 100 μm. **B** Representative multiplex immunofluorescence images of CD8 + tumor-infiltrating lymphocytes (TILs) and epithelial cells using antibodies against CD8 (red) and AE1/AE3 (green), and 4',6-diamidino-2-phenylindole (DAPI) (light blue), in GC and adjacent tissues from the CD8_IHC LOW, MID, and HIGH groups (from left to right). Scale bars, 100 μm. **C** Histograms showing the percentage of CD8 + TILs across 157 GC cases (left) and 34 single-cell RNA sequencing (scRNA-seq) cases (right). The percentage of CD8 + TILs refers to the proportion of CD8 + TILs among total tumor cells within a field at 200 × magnification. The gray dotted line is the reference line separating the LOW, MID, and HIGH groups. **D** Recurrence-free survival analysis (performed using the Kaplan–Meier plotter) of patients with GC (*n* = 157; LOW group = 37, MID group = 81, and HIGH group = 39). Statistical significance was set at *p* < 0.05 (ns: *p* ≥ 0.05, ∗ : *p* < 0.05, ∗∗ : *p* < 0.01, ∗  ∗  ∗: *p* < 0.001, and ∗  ∗  ∗  ∗ : *p* < 0.0001). **E** Overall survival analysis (performed using the Kaplan–Meier plotter) of patients (n = 157; LOW group = 37, MID group = 81, and HIGH group = 39). Statistical significance was set at *p* < 0.05 (ns: *p* ≥ 0.05, ∗ : *p* < 0.05, ∗  ∗ : *p* < 0.01, ∗  ∗  ∗ : *p* < 0.001, and ∗  ∗  ∗  ∗ : *p* < 0.0001)

We performed immunohistochemical staining with an anti-CD8 antibody on samples from 157 cases of GC (Fig. 1A). The field of view was selected at the tumor margin to include the stroma in the tumor’s advanced region (Fig. 1B). Epstein–Barr virus (EBV)-positive cases were diagnosed using Epstein–Barr-encoded RNA in situ hybridization (Figure S1A–B). We created a histogram to illustrate the percentage of CD8 + TILs across 157 GC cases. Based on the distribution, the cases were divided into groups with 0%–10%, 11%–21%, and ≥ 22% CD8 + T-cell infiltration, which were classified as the LOW, MID, and HIGH groups, respectively (Fig. 1C). Of the 157 GC samples, 34 underwent scRNA-seq analysis and were categorized into 21 in the LOW group, 7 in the MID group, and 6 in the HIGH group (Fig. 1C). We investigated the correlation between the degree of CD8 + T-cell infiltration and clinicopathological features, revealing that the MID group was associated with an age < 67 years (*p* = 0.0235), a lower T stage (*p* = 0.0059), a lower N stage (*p* = 0.0072) and a lower Tumor, Node, Metastasis (TNM) stage (*p* = 0.0006) (Table S1). We examined the prognostic value of each group using Kaplan–Meier curves. The HIGH and LOW groups had significantly lower RFS (*p* = 0.0438) and OS (*p* = 0.0002) than the MID group, but no significant differences were observed between the HIGH and LOW groups (Fig. 1D–E). When EBV-positive cases were separated from other cases in the HIGH group, the RFS and OS of the EBV-positive HIGH group showed differences compared to the other HIGH groups, although these differences were not statistically significant. These findings are consistent with a trend toward a better prognosis in the EBV-positive group, as previously reported (Figures S1C–D) [11]. Additionally, we performed multivariate analyses based on clinicopathological characteristics (Table S2). Multivariate Cox regression analysis for sex, age, T stage, N stage, pathological stage, and the MID group versus the LOW and HIGH groups confirmed that pathological stage and the degree of CD8 + T-cell infiltration in the MID group were independent prognostic factors for OS (hazard ratio = 0.1306, 95% confidence interval: 0.04142–0.3912, *p* = 0.0003; and hazard ratio = 0.3581, 95% confidence interval: 0.1287–0.9182,* p* = 0.0377 respectively).

### Variations in immune cell composition and *IFNG* expression within the GC TIME

**Fig. 2:**
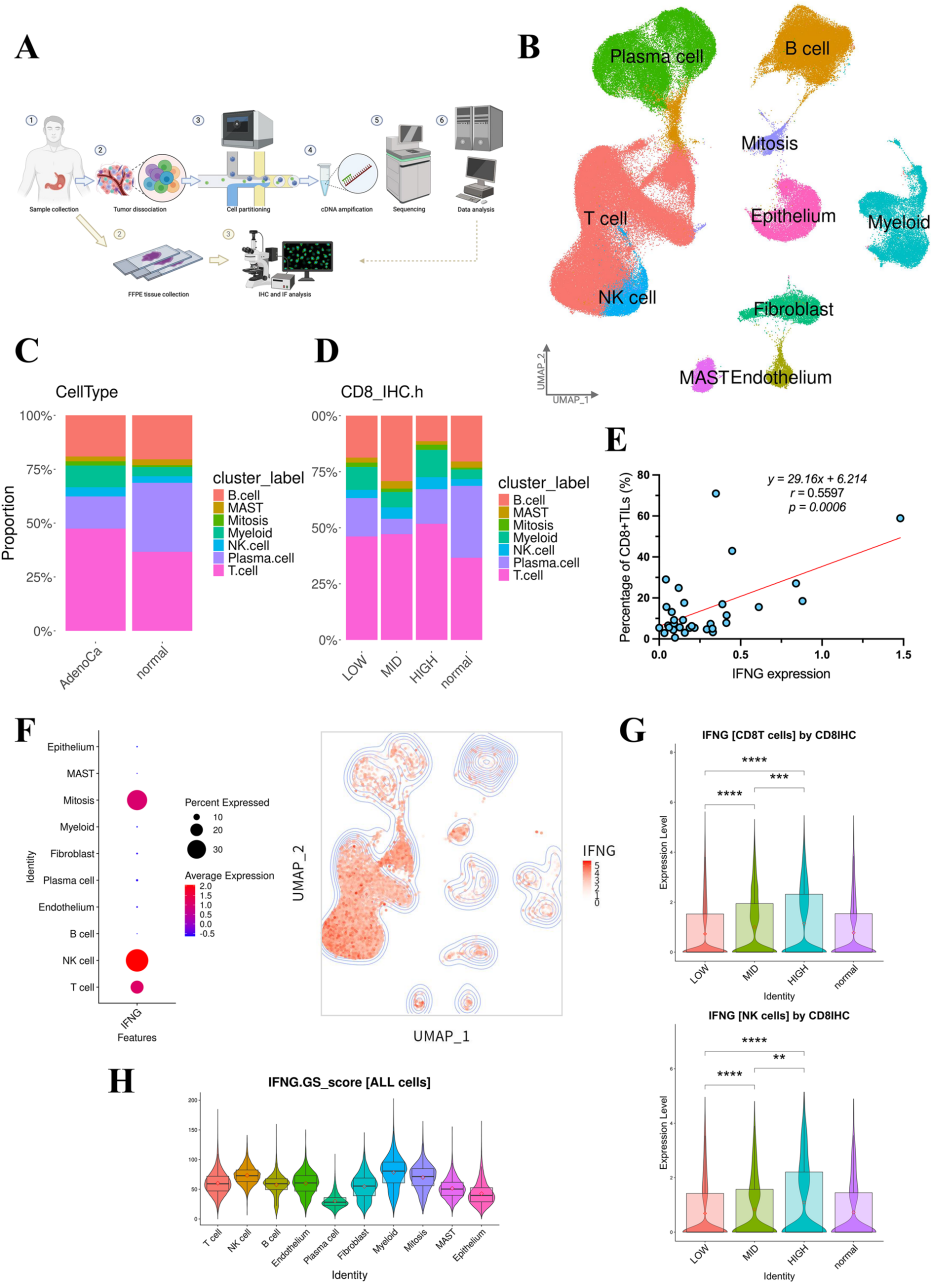
Variations in immune cell composition and *IFNG* expression within the GC tumor immune microenvironment. **A** Schematic illustration of the experimental workflow in our study. **B** Uniform manifold approximation and projection (UMAP) plot showing 10 color-coded major cell types, based on canonical marker genes. **C** Bar plots showing the proportions of seven immune cell clusters by the tissue source type. **D** Bar plots showing the proportions of seven immune cell clusters across four groups (HIGH, MID, LOW, and normal). **E** Scatter plot of CD8 + T-cell percentage and *IFNG* expression in GC cases. The regression equation and *p* value are displayed in the upper right corner. **F** Dot plot showing *IFNG* average expression in all cell clusters (left). The feature plot shows the *IFNG* average expression in UMAP (right). **G** Violin plots showing *IFNG* average expression in the CD8 + T-cells (top) and natural killer (NK) cells (bottom) across four groups (HIGH, MID, LOW, and normal). Statistical significance was set at *p* < 0.05 (ns: *p* ≥ 0.05, ∗ : *p* < 0.05, ∗  ∗ : *p* < 0.01, ∗  ∗  ∗ : *p* < 0.001, and ∗  ∗  ∗  ∗ : *p* < 0.0001). **H** Violin plots showing the IFNG gene set (IFNG.GS) score for all cell clusters

To explore the differences in the TIME based on the degree of CD8 + T-cell infiltration, we performed scRNA-seq on 41 human samples, including 34 from GC tumor tissues and 7 from normal tissues (Fig. 2A). The clinical characteristics of the patients associated with these samples were recorded (Table S3). After quality control and normalization, our scRNA-seq dataset included 199,609 cells (Fig. S2A). We used data integration to reduce batch effects, performed principal coordinate analysis on the integrated data, and applied graph-based clustering to identify distinct cell populations (Fig. S2B–C). Based on the differentially expressed genes (DEGs) and canonical marker expression in each cluster, we identified 10 major cell types (Figs. S2D and 2B). The percentage of each immune cell was calculated among *CD45*-expressing immune cells, and higher numbers of T-cells and myeloid cells were observed in the tumors compared with the normal tissues (Fig. 2C).

As shown in Fig. 1, we used CD8 staining of formalin-fixed paraffin-embedded specimens to classify the cases into HIGH, MID, and LOW groups based on CD8 + T-cell infiltration. The HIGH group showed a higher proportion of T-cells and myeloid cells compared with the MID group (Fig. 2D). Furthermore, after extracting all T-cells and NK cells and examining the proportions of CD4 + T-cells, CD8 + T-cells, and NK cells, we confirmed that the HIGH group had a higher proportion of CD8 + T-cells compared to the MID group, while the proportion of NK cells remained unchanged (Fig. S2E–F). We found that *IFNG* expression in the tumor environment correlated with the proportion of CD8 + TILs, even when excluding EBV-positive cases (Figs. 2E and S2G). *IFNG* was strongly expressed in T-cells and NK cells, mainly CD8 + TILs and NK cells (Figs. 2F and S2H), in which its expression increased stepwise across the groups (Fig. 2G). Interferon gamma (IFN-γ) is an inflammatory cytokine that activates T-cells and myeloid cells [12, 13]. Drawing from previous reports, we referenced a gene set influenced by the IFNG Gene Set (IFNG.GS) score to assess individual immune cell responsiveness to IFNG [13]. Among immune cells, the IFNG.GS score was high in T-cells, NK cells, myeloid cells, and B-cells (Fig. 2H). Overall, these results indicate that *IFNG* expression increases stepwise with the degree of CD8 + T-cell infiltration. Additionally, T-cells and myeloid cells, which comprise higher proportions of immune cells in the HIGH group, appear to be significantly associated with *IFNG* expression.

### Heterogeneity in the abundance and functionality of CD8 + TILs

**Fig. 3:**
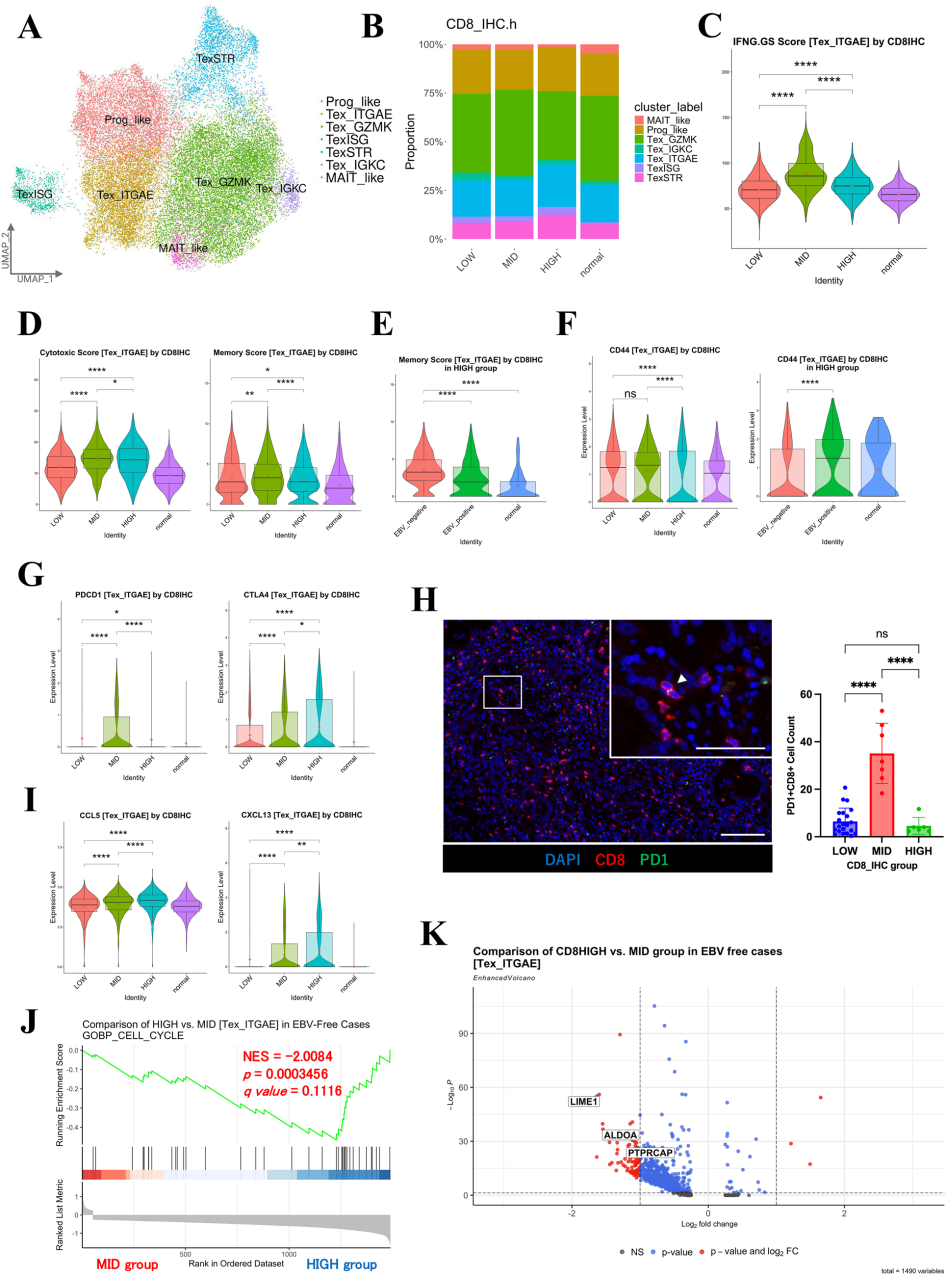
Heterogeneity in the abundance and functionality of CD8 + TILs. **A** UMAP plot showing seven color-coded subtype clusters of CD8 + TILs, based on representative genes. **B** Bar plots showing the proportions of seven subtype clusters of CD8 + TILs across four groups (HIGH, MID, LOW, and normal). **C** Violin plots showing IFNG.GS scores for the Tex_ITGAE subtype across four groups (HIGH, MID, LOW, and normal). Statistical significance was set at *p* < 0.05 (ns: *p* ≥ 0.05, ∗ : *p* < 0.05, ∗  ∗ : *p* < 0.01, ∗  ∗  ∗ : *p* < 0.001, and ∗  ∗  ∗  ∗ : *p* < 0.0001). **D** Violin plots showing the cytotoxic scores for the Tex_ITGAE subtype across four groups (HIGH, MID, LOW, and normal). Statistical significance was set at *p* < 0.05 (ns: *p* ≥ 0.05, ∗ : *p* < 0.05, ∗  ∗ : *p* < 0.01, ∗  ∗  ∗ : *p* < 0.001, and ∗  ∗  ∗  ∗ : *p* < 0.0001). **E** Violin plots showing the memory scores for the Tex_ITGAE subtype across four groups (HIGH, MID, LOW, and normal; left) and HIGH group (EBV-positive, EBV-negative, and normal; right). Statistical significance was set at *p* < 0.05 (ns: *p* ≥ 0.05, ∗ : *p* < 0.05, ∗  ∗ : *p* < 0.01, ∗  ∗  ∗ : *p* < 0.001, and ∗  ∗  ∗  ∗ : *p* < 0.0001). **F** Violin plots showing *CD44* expression in the Tex_ITGAE subtype across four groups (HIGH, MID, LOW, and normal; left), and three HIGH groups (EBV-positive, EBV-negative, and normal; right). Statistical significance was set at *p* < 0.05 (ns: *p* ≥ 0.05, ∗ : *p* < 0.05, ∗  ∗ : *p* < 0.01, ∗  ∗  ∗ : *p* < 0.001, and ∗  ∗  ∗  ∗ : *p* < 0.0001). **G** Violin plots showing *PDCD1* (left) and *CTLA4* (right) expression in the Tex_ITGAE subtype across four groups (HIGH, MID, LOW, and normal). Statistical significance was set at *p* < 0.05 (ns: *p* ≥ 0.05, ∗ : *p* < 0.05, ∗  ∗ : *p* < 0.01, ∗  ∗  ∗ : *p* < 0.001, and ∗  ∗  ∗  ∗ : *p* < 0.0001). **H** Representative multiplex immunofluorescence images of PD1 + CD8 + TILs using antibodies against CD8 (red), PD1 (green), and DAPI (light blue), in GC and adjacent tissues from the MID group (right). Arrowheads indicate PD1 + CD8 + cells. Scale bars, 100 μm and 50 μm (low and high magnification, respectively). Quantification bar plot of PD1 + CD8 + cell counts across three groups (HIGH, MID, and LOW; left). Statistical significance was set at *p* < 0.05 (ns: *p* ≥ 0.05, ∗ : *p* < 0.05, ∗  ∗ : *p* < 0.01, ∗  ∗  ∗ : *p* < 0.001, and ∗  ∗  ∗  ∗ : *p* < 0.0001). **I** Violin plots showing *CCL5* (left) and *CXCL13* (right) expression in the Tex_ITGAE subtype across four groups (HIGH, MID, LOW, and normal). Statistical significance was set at *p* < 0.05 (ns: *p* ≥ 0.05, ∗ : *p* < 0.05, ∗  ∗ : *p* < 0.01, ∗  ∗  ∗ : *p* < 0.001, and ∗  ∗  ∗  ∗ : *p* < 0.0001). **J** Gene set enrichment analysis results comparing the HIGH and MID groups, showing enrichment of “GOBP_CELL_CYCLE”-associated gene sets in the Tex_ITGAE subtype for EBV-negative cases. K. Volcano plot comparing the HIGH and MID groups in the Tex_ITGAE subtype for EBV-negative cases. The red dots in the volcano plot represent genes with a *p* value < 0.05 and a fold change (FC) > 1.0

To elucidate the heterogeneity of CD8 + TILs and how their functions change in each environment, we extracted CD8 + T-cell clusters and analyzed them in detail. After re-clustering, we identified seven subclusters (Figs. S3A–C and 3A): progenitor-like T-cells (Prog_like), exhausted T-cells (Tex) expressing *ITGAE* (Tex_ITGAE), Tex expressing *GZMK* (Tex_GZMK), Tex expressing *IGKC* (Tex_IGKC), Tex expressing stress-related genes (TexSTR), Tex expressing interferon-stimulated genes (TexISG), and mucosal-associated invariant T-cell-like T-cells (MAIT_like), based on previously reported genetic markers [14, 15]. The proportions of the clusters did not vary significantly among the different groups (Fig. 3B).

To investigate the functional state of the CD8 + TILs in antitumor immunity, we analyzed gene expression in the Tex_ITGAE subcluster, which has the highest cytotoxic score (Fig. S3D). The HIGH group showed higher *IFNG* expression than the MID group, but their CD8 + T-cells were less *IFNG*-responsive and had lower cytotoxicity and memory scores (Fig. 3C–D). However, memory scores were significantly higher in EBV-negative cases among the HIGH group (Fig. 3E). *CD44*, an activation marker, showed the lowest expression in the HIGH group (Fig. 3F) [16]. Similarly, *PDCD1*, an immune checkpoint gene, was downregulated in the HIGH group, whereas *CTLA4* was upregulated (Fig. 3G). Within the HIGH group, the analysis was divided by EBV positivity, which did not affect the overall landscape of low *PDCD1* and high *CTLA4* expression (Fig. S3E). Additionally, analysis of *TIGIT* and *LAG3* expression revealed that, compared to the MID group, the HIGH group showed higher expression of *TIGIT* and lower expression of *LAG3* (Fig. S3F). Furthermore, immunofluorescence staining confirmed that the number of PD1 + CD8 + T-cells was lower in the HIGH group than the MID group (Fig. 3H).

We next used CellChat to determine which chemokines were the most potent CD8 + T-cell inducers in the TIME. We found that the CCL5 (RANTES) signal pathway was highly ranked, with the highest expression in the EBV-negative HIGH group (Fig. S3G–I and 3I). CCL5 is more effective at inducing T-cell chemotaxis than IFNG itself [17]. Furthermore, *CXCL13* was most highly expressed in the EBV-negative HIGH group (Fig. 3I (Fig. S3I), whereas *CXCR5* expression in B-cells was highest in the MID group (Fig. S3J). The CXCL13–CXCR5 axis is involved in ectopic or tertiary lymphoid structure (TLS) formation [18].

To understand the decreased CD8 + T-cell activity in the HIGH group, we conducted gene set enrichment analysis (GSEA). Upon excluding EBV-positive cases, the HIGH group had significantly lower expression of cell cycle-related genes than the MID group (normalized enrichment score =  − 2.0084, *p* = 0.0003456, *q* = 0.1116) (Fig. 3J). Our volcano plot analysis revealed significant downregulation of Lck-interacting molecule 1 (*LIME1*), PTPRC-associated protein (*PTPRCAP*), and aldolase A (*ALDOA*) (Fig. 3K). Both LIME1 and PTPRCAP play important roles in TCR signaling and are required for T-cell activation [19, 20]. ALDOA is a glycolytic enzyme important in energy generation; it is involved in various cellular functions, such as cell shape maintenance and motility [21]. Thus, our data suggest that CD8 + TILs in the HIGH group recruit surrounding T-cells through chemokine signaling, thereby increasing cell count. However, these CD8 + T-cells show diminished cell division, downregulated TCR-related gene expression, and reduced glucose metabolism functionality.

### TCR repertoire diversity in different groups of CD8 + TILs

**Fig. 4:**
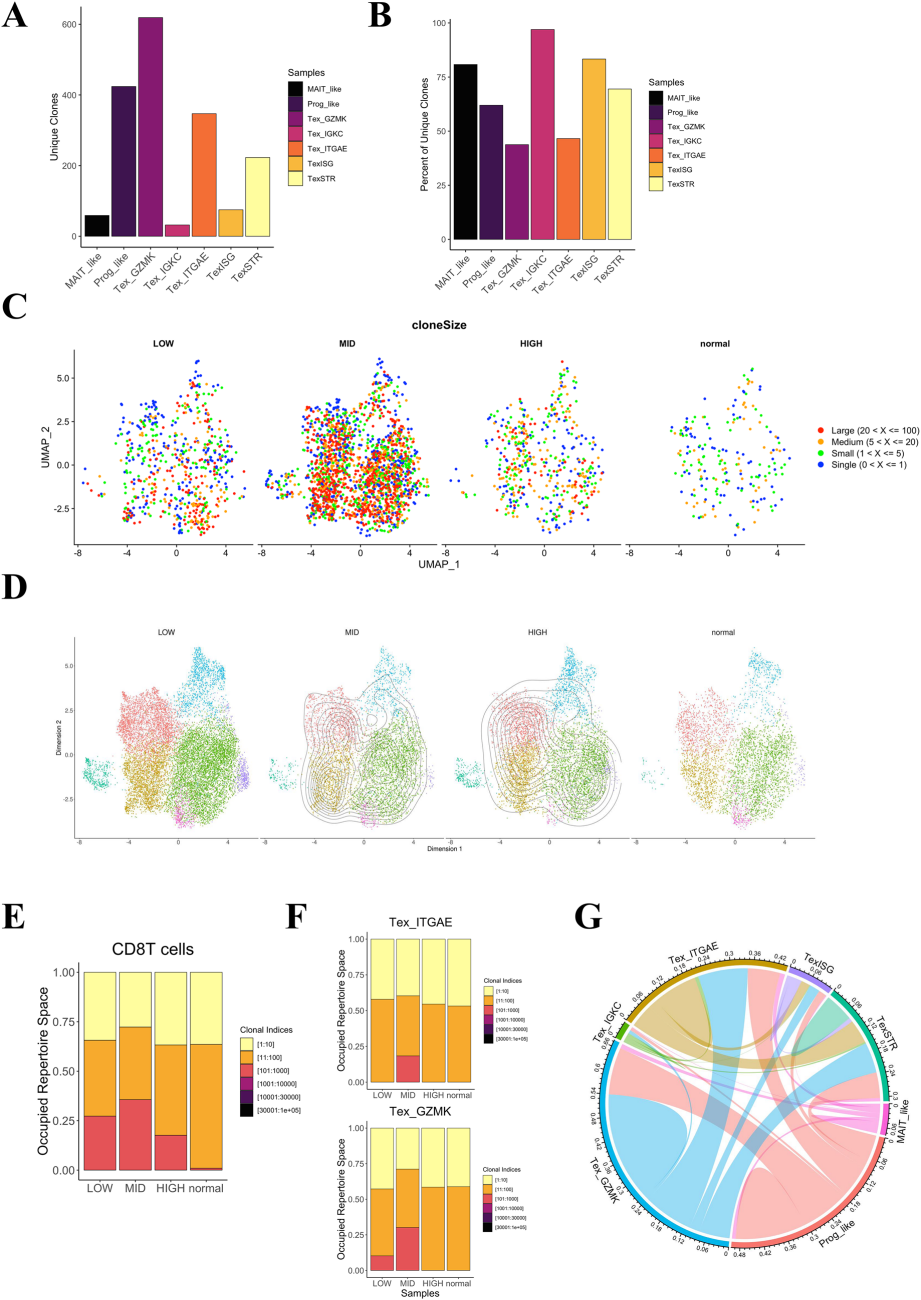
TCR repertoire diversity of different groups of CD8 + TILs. **A** Bar plots showing the unique clone counts of seven subtype clusters of CD8 + TILs across four groups (HIGH, MID, LOW, and normal). **B** Bar plots showing the percentage of unique clones within seven subtype clusters of CD8 + TILs across four groups (HIGH, MID, LOW, and normal). **C** UMAP plot showing the clone size of the CD8 + TILs across four groups (HIGH, MID, LOW, and normal). The UMAP plot shows the number of cells used for single-cell T-cell receptor sequencing (scTCR-seq): 471 cells in the HIGH group, 2063 cells in the MID group, 623 cells in the LOW group, and 203 cells in the normal group. **D** UMAP contour plot showing the clone frequency distribution of the CD8 + TILs across four groups (HIGH, MID, LOW, and normal). The background UMAP plot shows the number of cells used for scRNA-seq: 6477 cells in the HIGH group, 5359 cells in the MID group, 13,785 cells in the LOW group, and 4746 cells in the normal group. **E** Bar plots showing the proportions of occupied repertoire spaces of CD8 + TILs across four groups (HIGH, MID, LOW, and normal). **F** Bar plots showing the proportions of occupied repertoire spaces for the Tex_ITGAE (top) and Tex_GZMK (bottom) subtypes across four groups (HIGH, MID, LOW, and normal). **G** Chord diagram showing the clonal relationships between seven subtype clusters of CD8 + TILs

To assess whether TCR signaling is dysfunctional in the HIGH group, we analyzed scTCR-seq data from seven samples coupled with our scRNA-seq data [22]: two each from the HIGH, MID, and LOW groups, and one control sample (Table S3). None of the cases were EBV-positive. First, we examined the total number and percentage of unique clones to characterize the TCR sequences into clusters of CD8 + T-cells (Fig. 4A–B). The total number of unique clones was high in the Prog_like, Tex_ITGAE, and Tex_GZMK groups, with the highest number of unique clones in Tex_GZMK. The percentage of unique clones was low in the Tex_ITGAE and Tex_GZMK groups, which suggests that clonality is high. Therefore, we visualized the number of clones within each cluster across four groups (HIGH, MID, LOW, and normal) to identify the groups in which the clones were expanding (Fig. 4C). The MID group had the most clones, which were widely distributed in the Prog_like, Tex_ITGAE, and Tex_GZMK clusters. Additionally, by employing the clonalOverlay function to visualize the clonality transition within each cluster across different groups [23], we discovered that the Tex_ITGAE cluster exhibited lower clonal expansion in the HIGH group than in the MID group (Fig. 4D). The occupied CD8 + T-cell repertoire spaces showed greater clonality in the MID group than in the HIGH and LOW groups (Fig. 4E). This was also observed for the Tex_ITGAE and Tex_GZMK clusters (Fig. 4F). Finally, we examined the relative number of clones shared in each cluster and found that shared clones in Prog_like, Tex_ITGAE, and Tex_GZMK accounted for the majority (Fig. 4G). Briefly, TCR repertoire analysis showed decreased clonality in the HIGH group compared with the MID group.

### Characterization of myeloid cell dynamics and pathway interactions

**Fig. 5:**
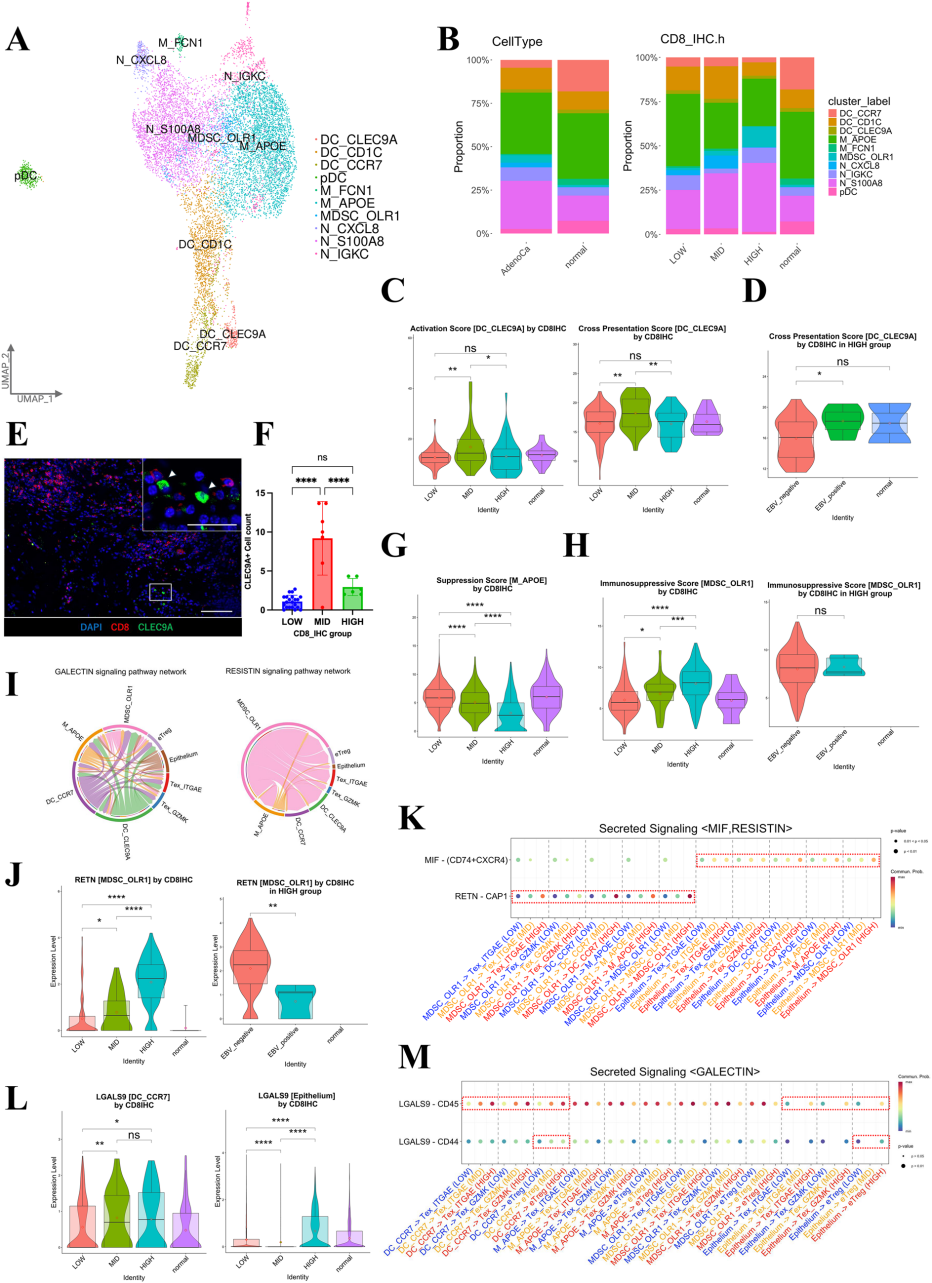
Characterization of the dynamics and pathway interactions of myeloid cells. **A** UMAP plot showing 10 color-coded subtype clusters of myeloid cells based on representative genes. **B** Bar plots showing the proportions of 10 subtype clusters of myeloid cells by the tissue source type (left) and in four groups (HIGH, MID, LOW, and normal; right). **C** Violin plots showing the activation scores (left) and cross-presentation scores (right) for the DC_CLEC9A subtype across four groups (HIGH, MID, LOW, and normal). Statistical significance was set at *p* < 0.05 (ns: *p* ≥ 0.05, ∗ : *p* < 0.05, ∗  ∗ : *p* < 0.01, ∗  ∗  ∗ : *p* < 0.001, and ∗  ∗  ∗  ∗ : *p* < 0.0001). **D** Violin plots showing the cross presentation scores of the DC_CLEC9A subtype across three HIGH groups (EBV-positive, EBV-negative, and normal; right). Statistical significance was set at *p* < 0.05 (ns: *p* ≥ 0.05, ∗ : *p* < 0.05, ∗  ∗ : *p* < 0.01, ∗  ∗  ∗ : *p* < 0.001, and ∗  ∗  ∗  ∗ : *p* < 0.0001). **E** Representative multiplex immunofluorescence images of CD8 + and CLEC9A + cells using antibodies against CD8 (red) and CLEC9A (green), and DAPI (light blue), in GC and adjacent tissues from the MID group. Arrowheads indicate CLEC9A + cells. Scale bars, 100 μm and 50 μm (low and high magnification, respectively).Statistical significance was set at *p* < 0.05 (ns: *p* ≥ 0.05, ∗ : *p* < 0.05, ∗  ∗ : *p* < 0.01, ∗  ∗  ∗ : *p* < 0.001, and ∗  ∗  ∗  ∗ : *p* < 0.0001). **F** Quantification bar plot of the CLEC9A + cell counts across three groups (HIGH, MID, and LOW). Statistical significance was set at *p* < 0.05 (ns: *p* ≥ 0.05, ∗ : *p* < 0.05, ∗  ∗ : *p* < 0.01, ∗  ∗  ∗ : *p* < 0.001, and ∗  ∗  ∗  ∗ : *p* < 0.0001). **G** Violin plots showing the suppression scores of the M_APOE subtype across four groups (HIGH, MID, LOW, and normal). Statistical significance was set at *p* < 0.05 (ns: *p* ≥ 0.05, ∗ : *p* < 0.05, ∗  ∗ : *p* < 0.01, ∗  ∗  ∗ : *p* < 0.001, and ∗  ∗  ∗  ∗ : *p* < 0.0001). **H** Violin plots showing the immunosuppressive scores of the MDSC_OLR1 subtype across four groups (HIGH, MID, LOW, and normal; left) and three HIGH groups (EBV-positive, EBV-negative, and normal; right). Statistical significance was set at *p* < 0.05 (ns: *p* ≥ 0.05, ∗ : *p* < 0.05, ∗  ∗ : *p* < 0.01, ∗  ∗  ∗ : *p* < 0.001, and ∗  ∗  ∗  ∗ : *p* < 0.0001). **I** Chord diagram showing the secreted signaling pathways of GALECTIN (left) and RESISTIN (right) among eight subtype clusters: M_APOE, MDSC_OLR1, DC_CLEC9A, DC_CCR7, eTreg, Tex_ITGAE, Tex_GZMK, and epithelial cells, using CellChat. **J** Violin plots showing *RETN* expression in the MDSC_OLR1 subtype across four groups (HIGH, MID, LOW, and normal; left) and three HIGH groups (EBV-positive, EBV-negative, and normal; right). Statistical significance was set at *p* < 0.05 (ns: *p* ≥ 0.05, ∗ : *p* < 0.05, ∗  ∗ : *p* < 0.01, ∗  ∗  ∗ : *p* < 0.001, and ∗  ∗  ∗  ∗ : *p* < 0.0001). **K** Dot plots showing the secreted signaling pathways (MIF and RESISTIN) in MDSC_OLR1 and epithelial cells across three groups (HIGH, MID, and LOW) using CellChat. **L** Violin plots showing *LGALS9* expression in the DC_CCR7 (left) and epithelial cells (right) across four groups (HIGH, MID, LOW, and normal). Statistical significance was set at *p* < 0.05 (ns: *p* ≥ 0.05, ∗ : *p* < 0.05, ∗  ∗ : *p* < 0.01, ∗  ∗  ∗ : *p* < 0.001, and ∗  ∗  ∗  ∗ : *p* < 0.0001). M. Dot plots showing the secreted signaling pathways (GALECTIN) from DC_CCR7, MDSC_OLR1, and epithelial cells across three groups (HIGH, MID, and LOW) using CellChat

We next analyzed myeloid cell clusters in detail. As a result of re-clustering, 10 subclusters were identified (Figs. S4A–B and 5A): dendritic cells (DCs) expressing *CCR7* (DC_CCR7), DCs expressing *CD1C* (DC_CD1C), DCs expressing *CLEC9A* (DC_CLEC9A), macrophages expressing *APOE* (M_APOE), macrophages expressing *FCN1* (M_FCN1), myeloid-derived suppressor cells (MDSCs) expressing *OLR1* (MDSC_OLR1), neutrophils expressing *CXCL8* (N_CXCL8), neutrophils expressing *IGKC* (N_IGKC), neutrophils expressing *S100A8* (N_S100A8), and plasmacytoid DCs (pDC), as described in previous reports [24–26]. We observed increased percentages of neutrophils and MDSCs in the tumor and in the HIGH group compared with the normal tissue and MID group, respectively, whereas the DC_CLEC9A percentages remained unchanged (Fig. 5B). Based on previous reports, we identified DC_CLEC9A as conventional type 1 DCs (cDC1s) that present antigens to CD8 + T-cells [24]. HIGH group cDC1s showed reduced IFNG-responsiveness, activity, and cross-presentation scores compared with MID group cDC1s, with a significant decrease in EBV-negative cases (Figs. S4C–D and 5C–D). Immunofluorescence staining revealed the highest number of cDC1s in the tumor margins of the MID group, with close proximity to CD8 + cells (Fig. S4E and 5E–F). Analysis of immunosuppressive cells showed that M2-like macrophages (M_APOE) had a higher immunosuppression score in the LOW group than in the MID and HIGH groups (Fig. 5G). Conversely, MDSC_OLR1 had the highest immunosuppression score in the HIGH group, regardless of EBV positivity (Fig. 5H).

Analysis of the secreted signaling pathways between cell types in the HIGH group using CellChat revealed that the GALECTIN, RESISTIN, and MIF pathways were ranked the highest (Fig. S3G). The GALECTIN pathway was primarily sourced from myeloid cells and epithelial cells, whereas the RESISTIN pathway was sourced only from MDSCs (Fig. 5I). The RESISTIN pathway, involved in chronic inflammation by binding to CAP1 in monocytes and upregulating inflammatory cytokines, is primarily expressed in MDSCs (Fig. S4F) [27]. It is most highly expressed in the EBV-negative HIGH group, with strong interactions with DC_CCR7, M_APOE, and CD8 + TILs (Fig. 5J–K). Additionally, among the three groups, the HIGH group exhibited enhanced MIF–CD74 pathway signaling, which upregulates CXCL8 in T-cells and induces neutrophils (Fig. 5K) [28]. Galectin, a β-galactoside-binding lectin, binds to glycans on cell membranes and inhibits TCR and BCR signaling via CD45 [29, 30]. Galectin-9 binds to CD44, activates Smad3 through TGF-β receptor complexes, and enhances Treg cell stability and function [31]. LGALS9 is highly expressed in DC_CCR7 and epithelial cells in the HIGH group, suggesting a role in T-cell immunosuppression (Fig. 5L). Cells in the DC_CCR7 subcluster, which highly express *CCR7* and *LAMP3*, are recognized as mature dendritic cells enriched in immunoregulatory molecules (mregDCs) [24]. Our analysis showed enhanced LGALS9–CD45 signaling (from mregDCs and epithelial cells to CD8 + T-cells) and LGALS9-CD44 signaling (to effector Tregs [eTregs]) in the HIGH group (Fig. 5M).

Thus, the HIGH group had lower expression of markers associated with cDC1 antigen-presenting capacity and higher expression of those associated with the suppressive function of MDSCs than the LOW and MID groups. The GALECTIN pathway was identified as a candidate factor for the suppression of TCR signaling in CD8 + TILs.

### Analysis of eTregs and *CTLA4* expression across different CD8 + TIL groups

**Fig. 6:**
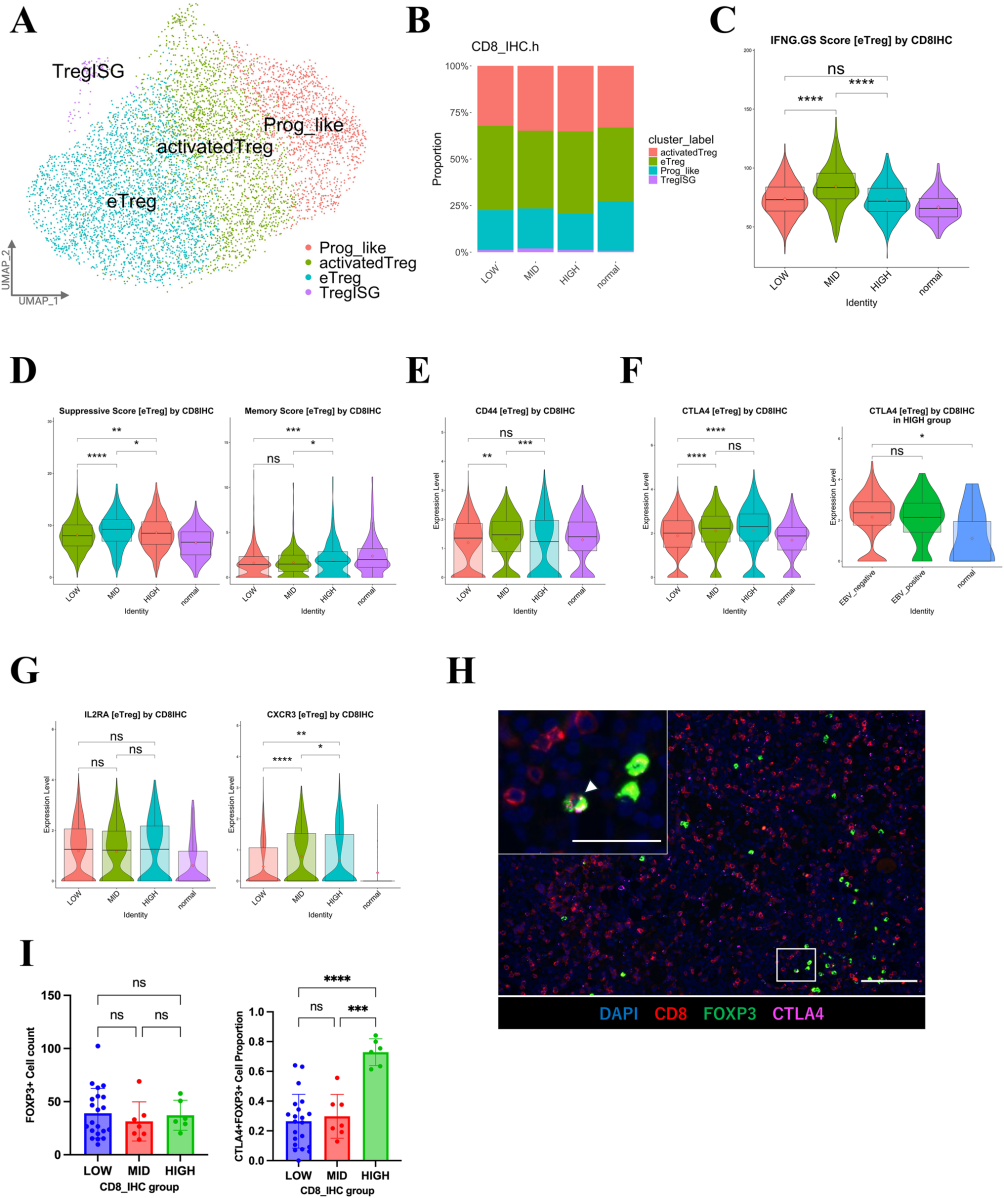
Analysis of Treg subclusters and *CTLA4* expression across different CD8 + TIL groups. **A** UMAP plot showing four color-coded subtype clusters of Tregs based on representative genes. **B** Bar plots showing the proportions of four subtype clusters of Tregs across four groups (HIGH, MID, LOW, and normal). **C** Violin plots showing the IFNG.GS scores of the eTreg subtype across four groups (HIGH, MID, LOW, and normal). Statistical significance was set at *p* < 0.05 (ns: *p* ≥ 0.05, ∗ : *p* < 0.05, ∗  ∗ : *p* < 0.01, ∗  ∗  ∗ : *p* < 0.001, and ∗  ∗  ∗  ∗ : *p* < 0.0001). **D** Violin plots showing the suppressive scores (left) and memory scores (right) of the eTreg subtype across four groups (HIGH, MID, LOW, and normal). Statistical significance was set at *p* < 0.05 (ns: *p* ≥ 0.05, ∗ : *p* < 0.05, ∗  ∗ : *p* < 0.01, ∗  ∗  ∗ : *p* < 0.001, and ∗  ∗  ∗  ∗ : *p* < 0.0001). **E** Violin plots showing *CD44* expression in the eTreg subtype across four groups (HIGH, MID, LOW, and normal). Statistical significance was set at *p* < 0.05 (ns: *p* ≥ 0.05, ∗ : *p* < 0.05, ∗  ∗ : *p* < 0.01, ∗  ∗  ∗ : *p* < 0.001, and ∗  ∗  ∗  ∗ : *p* < 0.0001). **F** Violin plots showing *CTLA4* expression in the eTreg subtype across four groups (HIGH, MID, LOW, and normal; left) and three HIGH groups (EBV-positive, EBV-negative, and normal; right). Statistical significance was set at *p* < 0.05 (ns: *p* ≥ 0.05, ∗ : *p* < 0.05, ∗  ∗ : *p* < 0.01, ∗  ∗  ∗ : *p* < 0.001, and ∗  ∗  ∗  ∗ : *p* < 0.0001). **G** Violin plots showing *IL2RA* (left) and *CXCR3* (right) expression in the eTreg subtype across four groups (HIGH, MID, LOW, and normal). Statistical significance was set at *p* < 0.05 (ns: *p* ≥ 0.05, ∗ : *p* < 0.05, ∗  ∗ : *p* < 0.01, ∗  ∗  ∗ : *p* < 0.001, and ∗  ∗  ∗  ∗ : *p* < 0.0001). **H** Representative multiplex immunofluorescence images of CD8 + and CTLA4 + FOXP3 + cells using antibodies against CD8 (red), FOXP3 (green), and CTLA4 (pink), and DAPI (light blue), in GC and adjacent tissues from the MID group. Arrowheads indicate CTLA4 + FOXP3 + cells. Scale bars, 100 μm and 50 μm (low and high magnification, respectively). **I** Quantification bar plot of FOXP3 + cell counts (left) and the CTLA4 + FOXP3 + cell proportion in the total FOXP3 + cells (right) across three groups (HIGH, MID, and LOW). The y-axis scale for the cell proportion in the figure ranges from 0.0 to 1.0 (0–100%). Statistical significance was set at *p* < 0.05 (ns: *p* ≥ 0.05, ∗ : *p* < 0.05, ∗  ∗ : *p* < 0.01, ∗  ∗  ∗ : *p* < 0.001, and ∗  ∗  ∗  ∗ : *p* < 0.0001)

After re-clustering of the Treg clusters, four subclusters were identified (Figs. S5A–C and 6A): progenitor-like Tregs (Prog_like), activated Tregs (activatedTreg), eTregs, and Tregs expressing interferon-stimulated genes (TregISG), as in previous reports [32]. The proportion of each cluster did not change significantly in the HIGH group compared with the MID group (Fig. 6B). As with CD8 + TILs, IFNG responsiveness was reduced in the HIGH group compared with the MID group, and the HIGH group had a lower Treg suppression score, but a high memory score (Fig. 6C–D). As in previous reports [32], *CTLA4* expression was highest in eTregs (Fig. S5D). *CD44* expression was low in the HIGH group, but *CTLA4* showed high expression, similar to the MID group (Fig. 6E–F). Furthermore, *CTLA4* expression tended to be higher in the EBV-negative HIGH group (Fig. 6F). The IL2 receptor is essential for regulating Treg homeostasis and suppressive activity [33]. *IL2RA* expression in eTregs did not differ significantly among the three groups (Fig. 6G). The chemokine receptor CXCR3, induced by IFN, promotes the migration of T-cells into inflammatory sites and the tumor microenvironment. *CXCR3*-expressing Tregs are abundant in tumors and inhibit CD8 + TIL activity by preferentially interacting with cDC1 [34]. *CXCR3* expression was highest in eTregs and maintained in the HIGH group, although at a lower level than in the MID group (Fig. S5E and 5G). Immunofluorescence staining showed a higher percentage of CTLA4 + Tregs in the HIGH group than the MID group, although the number of Tregs themselves was unchanged (Fig. 6H–I). Thus, although Treg activity was slightly lower in the HIGH group than in the MID group, the expression of the inhibitory receptor CTLA4 was higher, suggesting that it acts to suppress antitumor immunity.

### Immunosuppressive roles of fibroblasts in the GC microenvironment

**Fig. 7:**
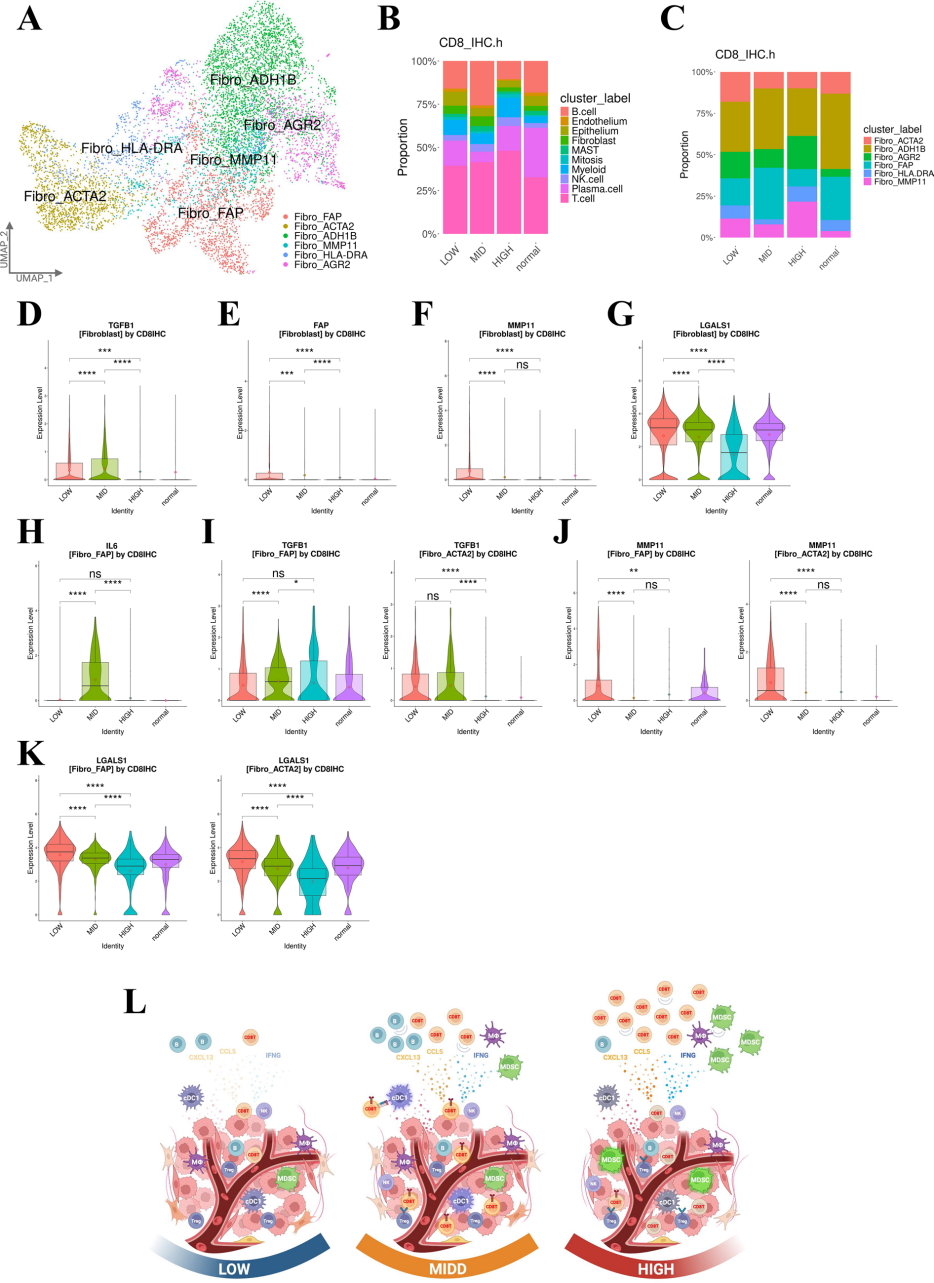
Immunosuppressive roles of fibroblasts in the GC microenvironment. **A** UMAP plot showing six color-coded subtype clusters of fibroblasts based on representative genes. **B** Bar plots showing the proportions of all clusters across four groups (HIGH, MID, LOW, and normal). **C** Bar plots showing the proportions of six subtype clusters of fibroblasts across four groups (HIGH, MID, LOW, and normal). **D** Violin plots showing *TGFB1* expression in all fibroblasts across four groups (HIGH, MID, LOW, and normal). Statistical significance was set at *p* < 0.05 (ns: *p* ≥ 0.05, ∗ : *p* < 0.05, ∗  ∗ : *p* < 0.01, ∗  ∗  ∗ : *p* < 0.001, and ∗  ∗  ∗  ∗ : *p* < 0.0001). **E** Violin plots showing *FAP* expression in all fibroblasts across four groups (HIGH, MID, LOW, and normal). Statistical significance was set at *p* < 0.05 (ns: *p* ≥ 0.05, ∗ : *p* < 0.05, ∗  ∗ : *p* < 0.01, ∗  ∗  ∗ : *p* < 0.001, and ∗  ∗  ∗  ∗ : *p* < 0.0001). **F** Violin plots showing *MMP11* expression in all fibroblasts across four groups (HIGH, MID, LOW, and normal). Statistical significance was set at *p* < 0.05 (ns: *p* ≥ 0.05, ∗ : *p* < 0.05, ∗  ∗ : *p* < 0.01, ∗  ∗  ∗ : *p* < 0.001, and ∗  ∗  ∗  ∗ : *p* < 0.0001). **G** Violin plots showing *LGALS1* expression in all fibroblasts across four groups (HIGH, MID, LOW, and normal). Statistical significance was set at *p* < 0.05 (ns: *p* ≥ 0.05, ∗ : *p* < 0.05, ∗  ∗ : *p* < 0.01, ∗  ∗  ∗ : *p* < 0.001, and ∗  ∗  ∗  ∗ : *p* < 0.0001). **H** Violin plots showing *IL6* expression in the Fibro_FAP subtype across four groups (HIGH, MID, LOW, and normal). Statistical significance was set at *p* < 0.05 (ns: *p* ≥ 0.05, ∗ : *p* < 0.05, ∗  ∗ : *p* < 0.01, ∗  ∗  ∗ : *p* < 0.001, and ∗  ∗  ∗  ∗ : *p* < 0.0001). **I** Violin plots showing *TGFB1* expression in the Fibro_FAP (left) and Fibro_ACTA2 subtype (right) across four groups (HIGH, MID, LOW, and normal). Statistical significance was set at *p* < 0.05 (ns: *p* ≥ 0.05, ∗ : *p* < 0.05, ∗  ∗ : *p* < 0.01, ∗  ∗  ∗ : *p* < 0.001, and ∗  ∗  ∗  ∗ : *p* < 0.0001). **J** Violin plots showing *MMP11* expression in the Fibro_FAP (left) and Fibro_ACTA2 subtype (right) across four groups (HIGH, MID, LOW, and normal). Statistical significance was set at *p* < 0.05 (ns: *p* ≥ 0.05, ∗ : *p* < 0.05, ∗  ∗ : *p* < 0.01, ∗  ∗  ∗ : *p* < 0.001, and ∗  ∗  ∗  ∗ : *p* < 0.0001). **K** Violin plots showing *LGALS1* expression in the Fibro_FAP (left) and Fibro_ACTA2 subtype (right) across four groups (HIGH, MID, LOW, and normal). Statistical significance was set at *p* < 0.05 (ns: *p* ≥ 0.05, ∗ : *p* < 0.05, ∗  ∗ : *p* < 0.01, ∗  ∗  ∗ : *p* < 0.001, and ∗  ∗  ∗  ∗ : *p* < 0.0001). **L** Schematic illustrations of the results for the LOW, MID, and HIGH groups

We re-clustered fibroblasts into six subclusters: Fibro_FAP, Fibro_ACTA2, Fibro_ADH1B, Fibro_MMP11, Fibro_HLA-DRA, and Fibro_AGR2 (Fig. S6A–B and 7A) [35, 36]. Based on previous studies, Fibro_FAP was classified as inflammatory cancer-associated fibroblast (iCAF)-like, Fibro_ACTA2 as myofibroblastic cancer-associated fibroblast (myCAF)-like, and Fibro_HLA-DRA and Fibro_AGR2 as antigen-presenting cancer-associated fibroblast (apCAF)-like (Fig. S6C) [37]. The proportion of fibroblasts among major cells was lower in the HIGH group compared to the LOW and MID groups (Fig. 7B). The MID group showed an increased proportion of Fibro_FAP, whereas the proportion of Fibro_ACTA2 was higher in the LOW group (Fig. 7C).

TGF-β, produced by cancer-associated fibroblasts (CAFs), directly suppresses anti-tumor immunity [38]. Fibroblast activation protein (FAP)-positive and galectin-1 (*LGALS1*)-positive CAFs have been shown to inhibit T-cell activation and infiltration within tumors [39, 40]. Therapies targeting matrix metalloproteinase 11 (MMP11) can promote cell-mediated immune responses, resulting in anti-tumor effects [41]. In this study, we observed higher *TGFB1* expression in fibroblasts from the MID and LOW groups compared to the HIGH group (Fig. 7D). Additionally, *FAP*, *MMP11*, and *LGALS1* expression in fibroblasts was highest in the LOW group (Fig. 7E–G). Furthermore, as iCAFs are known to produce cytokine IL6, which contributes to immunosuppression, we found that *IL6* and *TGFB1* expression in Fibro_FAP was highest in the MID group (Fig. 7H–I) [37]. *TGFB1* levels in Fibro_ACTA2 were elevated in both the LOW and MID groups (Fig. 7I). Notably, *MMP11* and *LGALS1* expression was elevated in both Fibro_FAP and Fibro_ACTA2 in the LOW group (Fig. 7J–K). Overall, these findings suggest that CAFs may contribute more significantly to immunosuppression in GC in the LOW and MID groups compared to the HIGH group.

## Discussion

We classified the tumor environment of GC samples based on CD8 + TIL abundance through scRNA-seq data analysis to elucidate the complexity and heterogeneity of the TIME. We found that the MID group had the best prognosis. Surprisingly, the HIGH group with abundant CD8 + TILs had a poor prognosis. Among the HIGH group, EBV-positive cases appeared to have a better prognosis, which was consistent with previous reports [11]. In this study, the LOW group demonstrated poor OS (Fig. 7L), likely due to lower antitumor activity owing to the absence of tumor antigens, as previously reported [42, 43]. Additionally, the present data suggest that M2-like macrophages and CAFs contribute more to immunosuppressive activity in the LOW and MID groups than in the HIGH group.

However, the cause of poor prognosis in the HIGH group remained unclear. Tex_ITGAE exhibited high levels of immune checkpoint and cytotoxic molecules, classifying it as an effector or resident memory cluster [44], and Tex_GZMK displayed a prominent memory signature [45]. Given the importance of effector and memory T-cells in the antitumor response, we focused on these clusters. Compared with the MID group, the HIGH group had CD8 + TILs with lower IFNG-responsiveness and activation marker expression, as well as lower levels of cell division and clonality, with the exception of the EBV-positive cases. We found that *PDCD1* and *LAG3* expression was significantly lower in the HIGH group compared to the MID group, contrasting with previous reports describing a gradual increase in PD1 and LAG3 expression under chronic antigen exposure [46]. Conversely, *TIGIT* and *CTLA4* were markedly upregulated in the HIGH group, consistent with their reported rise during the early phase of T-cell activation [47–49]. These findings suggest that the HIGH group comprises clusters of weakly activated bystander CD8 + T-cells, rather than tumor-antigen specific activated and exhausted CD8 + T-cells (Fig. 7L).

Previous reports indicate that galectins negatively regulate Lck activity in resting cells, suppress TCR clustering and signaling via CD45, and facilitate CTLA4 retention on the T-cell surface, resulting in growth arrest [29]. In this study, galectins were expressed in epithelial cells and mregDCs with the highest signal in the HIGH group. The GALECTIN pathway may contribute to the high *CTLA4* expression in low-activity CD8 + TILs in the HIGH group.

Our analysis revealed an increased proportion and enhanced suppressive function of MDSCs in the HIGH group. The RESISTIN and MIF pathways in MDSCs and epithelial cells were also enhanced; they may contribute to the chemotaxis of tumor-infiltrating immune cells. Additionally, cDC1s in the HIGH group had reduced activity and antigen-presenting capacity, which may be due to suppression by MDSCs and eTregs. eTreg activity was preserved in the HIGH group, with high *CTLA4* expression.

TLS have recently been found in various cancers, in which they are associated with a favorable prognosis [18]. The HIGH group had a lower proportion of B-cells and significantly lower *CXCR5* expression than the MID group, especially among EBV-negative cases. The GALECTIN pathway, which was enhanced in the HIGH group, also suppresses BCR signaling [30]. Taken together, our data suggest TLS formation is more efficient in the MID group (Fig. 7L).

Our study has redefined the TIME landscape in GC. However, in this study, only seven TCR cases were analyzed and the number of EBV-positive cases examined was limited; these limitations necessitate a larger dataset in future studies. Furthermore, it will be necessary to investigate whether similar patterns are observed in other cancer types. In future studies, the use of spatial transcriptomics would provide greater insight into the TIME [50].

In conclusion, the group with the highest abundance of CD8 + TILs in GC tumors exhibited poor survival prognosis, which may be attributable to the chemotaxis-driven infiltration of inactive CD8 + T-cells with low clonality. The suppressive signaling pathways in epithelial cells and immunosuppression by MDSCs and Tregs may further contribute to the inactivation of CD8 + TILs in the HIGH group.

## Supplementary Information

Below is the link to the electronic supplementary material.

Supplementary file1 (DOCX 104 KB)

Supplementary file2 (XLSX 11 KB)

Supplementary file3 (XLSX 11 KB)

Supplementary file4 (XLSX 12 KB)

Supplementary file5 (XLSX 13 KB)

Supplementary file6 (PDF 882 KB) Figure S1. Prognostic impact of the degree of CD8+ T-cell infiltration. A. Representative in situ hybridization image of Epstein–Barr-encoded RNA immunostaining of GC tissue from the HIGH (EBV) group. Scale bar, 100 μm. B. Representative multiplex immunofluorescence images of CD8+ TILs and epithelial cells using antibodies against CD8 (red) and AE1/AE3 (green), and DAPI (light blue), in GC and adjacent tissues from the HIGH (EBV) group. Scale bar, 100 μm. C. Recurrence-free survival analysis (performed using the Kaplan–Meier plotter) of patients with GC (n=39; HIGH (other) group=34, HIGH (EBV+) group=5). Statistical significance was set at p<0.05 (ns: p≥0.05, ∗: p<0.05, ∗∗: p<0.01, ∗∗∗: p<0.001, and ∗∗∗∗: p<0.0001). D. Overall survival analysis (performed using the Kaplan–Meier plotter) of patients (n=39; HIGH (other) group=34, HIGH (EBV+) group=5). Statistical significance was set at p<0.05 (ns: p≥0.05, ∗: p<0.05, ∗∗: p<0.01, ∗∗∗: p<0.001, and ∗∗∗∗: p<0.0001)

Supplementary file7 (PDF 1077 KB) Figure S2. Variations in immune cell composition and IFNG expression within the GC tumor immune microenvironment. A. UMAP plot showing the clustering of cells among all cells from 41 original samples. B. UMAP plot showing the clustering of cells by the tissue source type among all cells. C. UMAP plot showing 28 clusters among all cells from 34 resected tumor tissues and 7 normal tissues. D. Heatmap of the canonical marker genes in each major cell type in all cells. E. UMAP plot of T- cells and NK cells showing clustering of CD8+ T-cells (red), CD4+ T-cells (green), and NK cells (blue). The clustering was performed to visualize the distinct populations based on their gene expression profiles. F. Bar plots showing the proportions of CD8+ T-cells, CD4+ T-cells, and NK cells across the four groups (HIGH, MID, LOW, and normal). G. Scatter plot of CD8+ T-cell percentage and IFNG expression in patients with GC excluding EBV-positive cases. The regression equation and p value are displayed in the upper right corner. H. Violin plots showing IFNG expression in CD8+ T-cells, CD4+ T-cells, and NK cells

Supplementary file8 (PDF 1753 KB) Figure S3. Heterogeneity in the abundance and functionality of CD8+ TILs. A. UMAP plot showing the clustering of cells among CD8+ T-cells from 41 original samples. B. Heatmap of the top 10 genes among the differentially expressed genes (DEGs) in each subtype of CD8+ T-cells. C. Dot plots showing canonical marker gene expression in the CD8+ T-cell subtypes. D. Violin plots showing the cytotoxic scores of the CD8+ T-cell subtypes. Statistical significance was set at p<0.05 (ns: p≥0.05, ∗: p<0.05, ∗∗: p<0.01, ∗∗∗: p<0.001, and ∗∗∗∗: p<0.0001). E. Violin plots showing PDCD1 (left) and CTLA4 (right) expression in the Tex_ITGAE subtype across three HIGH groups (EBV-positive, EBV-negative, and normal). Individual points represent single cells. Statistical significance was set at p<0.05 (ns: p≥0.05, ∗: p<0.05, ∗∗: p<0.01, ∗∗∗: p<0.001, and ∗∗∗∗: p<0.0001). F. Violin plots showing TIGIT (left) and LAG3 (right) expression in the Tex_ITGAE subtype across four groups (HIGH, MID, LOW, and normal). Statistical significance was set at p<0.05 (ns: p≥0.05, ∗: p<0.05, ∗∗: p<0.01, ∗∗∗: p<0.001, and ∗∗∗∗: p<0.0001). G. Ranking of the top 10 ligand–receptor pairs in all cell clusters within the HIGH group using CellChat. H. Dot plots showing the secreted signaling pathways (CXCL and CCL) in the Tex_ITGAE and Tex_GZMK subtypes across three groups (HIGH, MID, and LOW) using CellChat. I. Violin plots showing CCL5 (left) and CXCL13 (right) expression in the Tex_ITGAE subtype across three HIGH groups (EBV-positive, EBV-negative, and normal). Statistical significance was set at p<0.05 (ns: p≥0.05, ∗: p<0.05, ∗∗: p<0.01, ∗∗∗: p<0.001, and ∗∗∗∗: p<0.0001). J. Violin plots showing CXCR5 expression by B-cells across four groups (HIGH, MID, LOW, and normal; left) and three HIGH groups (EBV-positive, EBV-negative, and normal; right). Statistical significance was set at p<0.05 (ns: p≥0.05, ∗: p<0.05, ∗∗: p<0.01, ∗∗∗: p<0.001, and ∗∗∗∗: p<0.0001)

Supplementary file9 (PDF 2381 KB) Figure S4. Characterization of the dynamics and pathway interactions of myeloid cells. A. UMAP plot showing the clustering of cells among myeloid cells from 41 original samples. B. Heatmap of the top 10 genes among the DEGs in each subtype of myeloid cells. C. Violin plots showing the IFNG.GS scores of the DC_CLEC9A subtype across four groups (HIGH, MID, LOW, and normal). Statistical significance was set at p<0.05 (ns: p≥0.05, ∗: p<0.05, ∗∗: p<0.01, ∗∗∗: p<0.001, and ∗∗∗∗: p<0.0001). D. Violin plots showing the IFNG.GS scores of the DC_CLEC9A subtype across three HIGH groups (EBV-positive, EBV-negative, and normal). Statistical significance was set at p<0.05 (ns: p≥0.05, ∗: p<0.05, ∗∗: p<0.01, ∗∗∗: p<0.001, and ∗∗∗∗: p<0.0001). E. Quantification bar plot of interaction counts between CLEC9A+ cells and CD8+ cells across three groups (HIGH, MID, and LOW). The interaction counts were defined as the presence of one or more CD8+ cells within a 20-μm radius centered on the CLEC9A+ cells. Statistical significance was set at p<0.05 (ns: p≥0.05, ∗: p<0.05, ∗∗: p<0.01, ∗∗∗: p<0.001, and ∗∗∗∗: p<0.0001). F. Violin plots showing RETN expression in the myeloid cell clusters

Supplementary file10 (PDF 2483 KB) Figure S5. Analysis of Treg subclusters and CTLA4 expression across different CD8+ TIL groups. A. UMAP plot showing the clustering of cells among Tregs from 41 original samples. B. Heatmap of the top 10 genes from the DEGs in each subtype in Tregs. C. Dot plots showing canonical marker gene expression in the Treg subtypes. D. Violin plots showing CTLA4 expression in the Treg subtypes. E. Violin plots showing CXCR3 expression in the Treg subtypes

Supplementary file11 (PDF 798 KB) Figure S6. Immunosuppressive roles of fibroblasts in the GC microenvironment. A. UMAP plot showing the clustering of cells among fibroblasts from 41 original samples. B. Heatmap of the top 10 genes from the DEGs in each subtype in fibroblasts. C. Heatmap of the canonical marker genes in each subtype in fibroblasts

## Data Availability

The scRNA-seq data generated in this study are available from the GEO database under accession code GSE271913. The remaining data are available within the manuscript, in the supplementary materials, or from the corresponding author upon reasonable request.
