# Supplementary material for: Tumor infiltration of inactive CD8 + T cells was associated with poor prognosis in Gastric Cancer
Source: Gastric Cancer. 2024 Dec 25;28(2):211–27. doi: 10.1007/s10120-024-01577-4 (PMC11842491; doi:10.1007/s10120-024-01577-4)
Supplement: Supplementary file 1 — Supplementary file1 (DOCX 104 KB) [file 10120_2024_1577_MOESM1_ESM.docx]

**Supplementary Materials and Methods**

**Human GC samples collection**

It was carried out based on the International Union for Cancer classification of tumors, lymph nodes, and metastases. All fresh specimens collected were obtained from the tumor site and adjacent normal tissue after surgical removal. The clinicopathological characteristics of patients are listed in Table S3.

**ScRNA-seq / scTCR-seq library preparation**

Freshly harvested tumor tissue and normal mucosa were isolated for genetic sequencing immediately after surgery. This experiment was performed based on the protocol published online (<http://dx.doi.org/10.17504/protocols.io.b2udqes6>). The scRNA-seq libraries were prepared using the Chromium Next GEM Single Cell 5' GEM Kit V2 (product number PN-1000244), Library Construction Kit (product number PN-1000190), and Next GEM Single Cell 5’ Gel Beads v2 (product number PN-1000264). All kits were supplied by 10x Genomics, Inc. (Pleasanton, CA, USA). Libraries were constructed following the protocols provided by the manufacturer. On average, approximately 5,000 live cells were used from each sample. The Single-cell TCR V(D)J sequencing (scTCR-seq) libraries were prepared using the Chromium Single Cell Human TCR Amplification Kit (product number PN-1000252) and Library Construction Kit (product number PN-1000190), also supplied by 10x Genomics, Inc. (Pleasanton, CA, USA). Library construction adhered to the manufacturer’s specified protocols. On average, approximately 2,000 live cells were used from each sample. These scRNA-seq samples were sequenced on an Illumina NovaSeq 6000 (Illumina, San Diego, California, USA) or DNBSEQ-G400 (MGI Tech, Shenzhen, China).

**ScRNA-seq / scTCR-seq data analysis**

From the sequence data, we removed cells with fewer than three expressed genes and fewer than 200 expressed genes at the time of object creation as cells of low quality. In addition, quality control was performed after the quality check based on the total number of genes, total UMI number, and mitochondrial RNA ratio, and cells with low quality (< 200 unique molecular identifiers/cell, > 25% mitochondrial genes) were excluded. We then normalized the data based on the expression level of each gene relative to the total gene expression level in the cell and found 2000 useful genes with high cell-to-cell variation in the dataset. We then used the “CellCycleScoring” and “SCTransform” functions to reduce the influence of cell cycle heterogeneity in the scRNA-seq data and also remove the influence of batch effects [1,2]. Furthermore, the identified doublet cells were filtered using the "DoubletFinder" function [3]. Principal component analysis was performed using the "RunPCA" function, clusters were estimated using the "FindNeighbor" function, and visualized using the uniform manifold approximation projection (UMAP) method with "FindClusters". After integrating the data, we used the "Harmony" function to adjust the data to mitigate batch effects of multiple scRNA-seq data and achieve biologically consistent cell clustering across different samples [4]. After each cluster was identified, extraction was performed, and among immune cells, only PTPRC (gene encoding CD45) positive cells were analyzed. Additionally, cell-cell interactions were assessed using “CellChat” [5]. For scTCR-seq data analysis, TCR sequencing data and scRNA-seq data of CD8+ T-cells were integrated using “scRepertoire” [6].

**Gene signatures**

To understand the functional characteristics of specific cell subtypes and biological states, we established a set of genes that characterize specific functions and cell types as gene signatures, and analyzed and evaluated them. The gene signatures are listed in Supplementary Table S4.

**Differentially expressed genes and pathway analysis**

Differentially expressed genes (DEGs) were analized by using a two-sided Wilcoxon rank-sum test with Bonferroni FDR collection. We conducted Gene Set Enrichment Analysis (GSEA) with the DEGs identified by "FindMarkers" tool for the cell subtypes [7].

**Immunohistochemistry and immunofluorescence**

The formalin-fixed paraffin-embedded (FFPE) samples were cut as 4-μm sections. These sections were deparaffined with xylene and ethanol. Endogenous peroxidase activity was blocked by methanol with 0.3% hydrogen peroxidase for 30 minutes. These slides were retrieved the antigen-epitopes using a microwave oven or pressure cooker for 20 min in Citrate Buffer (pH 5.9) or Tris-EDTA Buffer (pH 9.0). Blocking was performed for unspecific binding sites with PBS+3% BSA for 30 minutes. After that, these slides were incubated with primary antibodies at 4°C overnight. The primary antibodies were as follows: CD8 (Nichirei, Tokyo, Japan, # 413201), CD8a (53-6.7; eBioscience™, San Diego, USA, # 14-0081-82), CLEC9A (Proteintech, Rosemont, USA, #55451-1-AP), FOXP3 (FJK-16s; eBioscience™, San Diego, USA, # 14-5773-82), CTLA4 (Abcam, Cambridge, UK, #ab237712), PD1 (Abcam, Cambridge, UK, #ab137132), AE1, AE3 (Nichirei, Tokyo, Japan, # 412811) . The next day, these slides were rinsed and incubated with secondary antibody (DAKO/Agilent, Glostrup, Denmark, Rabbit, K4003, and Mouse, K4001) for 40 minutes at room temperature. We performed Nuclei counterstain with hematoxylin (Muto Pure Chemicals Co., Ltd, Tokyo, Japan, #30002). In immunohistochemistry, the staining was performed with a 3,3′-diaminobenzidine (DAB) kit (Sigma-Aldrich, Darmstadt, Germany, #D5537-5G). In immunofluorescence, these specimens were incubated with secondary antibodies in PBS+0.1%BSA for an hour at room temperature. Secondary antibodies for immunofluorescence were as follows: Donkey-anti-mouse-AF546, Donkey-anti-goat-AF647, Donkey-anti-rabbit-AF488 (Invitrogen, Massachusetts, USA, 1:200). The nuclei were stained with 4',6-diamidino-2-phenylindole (DAPI) (DOJINDO, Tokyo, Japan, 1:1000). Fluorescence images were acquired with an optical microscope (BZ-X800; Keyence), and counting was performed using Fiji [8]. Basically, we randomly selected three areas, including the advanced tumor, the tumor margin, and the stroma, counted CD8-positive cells and tumor cells at 200x magnification, and calculated the ratio of each. The average of these ratios was then calculated. Two pathologists independently performed this evaluation. In the histogram illustrating CD8+ TIL proportions among 157 cases, the MID group was defined as the middle 50% range centered on the median value (16.09), while the remaining cases were assigned to the HIGH and LOW groups. For EBV cases, diagnoses were confirmed by hospital pathologists and pathology researchers via Epstein-Barr encoded RNA (EBER) in situ hybridization (ISH).

**References**

[1] C. Hafemeister and R. Satija, “Normalization and variance stabilization of single-cell RNA-seq data using regularized negative binomial regression,” *Genome Biol*, vol. 20, no. 1, Dec. 2019, doi: 10.1186/S13059-019-1874-1.

[2] S. Nestorowa *et al.*, “A single-cell resolution map of mouse hematopoietic stem and progenitor cell differentiation,” *Blood*, vol. 128, no. 8, pp. e20–e31, Aug. 2016, doi: 10.1182/BLOOD-2016-05-716480.

[3] C. S. McGinnis, L. M. Murrow, and Z. J. Gartner, “DoubletFinder: Doublet Detection in Single-Cell RNA Sequencing Data Using Artificial Nearest Neighbors,” *Cell Syst*, vol. 8, no. 4, pp. 329-337.e4, Apr. 2019, doi: 10.1016/J.CELS.2019.03.003.

[4] I. Korsunsky *et al.*, “Fast, sensitive and accurate integration of single-cell data with Harmony,” *Nat Methods*, vol. 16, no. 12, pp. 1289–1296, Dec. 2019, doi: 10.1038/S41592-019-0619-0.

[5] S. Jin *et al.*, “Inference and analysis of cell-cell communication using CellChat,” *Nature Communications 2021 12:1*, vol. 12, no. 1, pp. 1–20, Feb. 2021, doi: 10.1038/s41467-021-21246-9.

[6] N. Borcherding and N. L. Bormann, “scRepertoire: An R-based toolkit for single-cell immune receptor analysis,” *F1000Research 2020 9:47*, vol. 9, p. 47, Jan. 2020, doi: 10.12688/f1000research.22139.1.

[7] A. Subramanian *et al.*, “Gene set enrichment analysis: a knowledge-based approach for interpreting genome-wide expression profiles,” *Proc Natl Acad Sci U S A*, vol. 102, no. 43, pp. 15545–15550, Oct. 2005, doi: 10.1073/PNAS.0506580102.

[8] J. Schindelin *et al.*, “Fiji: an open-source platform for biological-image analysis,” *Nat Methods*, vol. 9, no. 7, pp. 676–682, Jul. 2012, doi: 10.1038/NMETH.2019.
