# Supplementary figures and images for: Tumor infiltration of inactive CD8 + T cells was associated with poor prognosis in Gastric Cancer
Source: Gastric Cancer. 2024 Dec 25;28(2):211–27. doi: 10.1007/s10120-024-01577-4 (PMC11842491; doi:10.1007/s10120-024-01577-4)

Figure S1

A

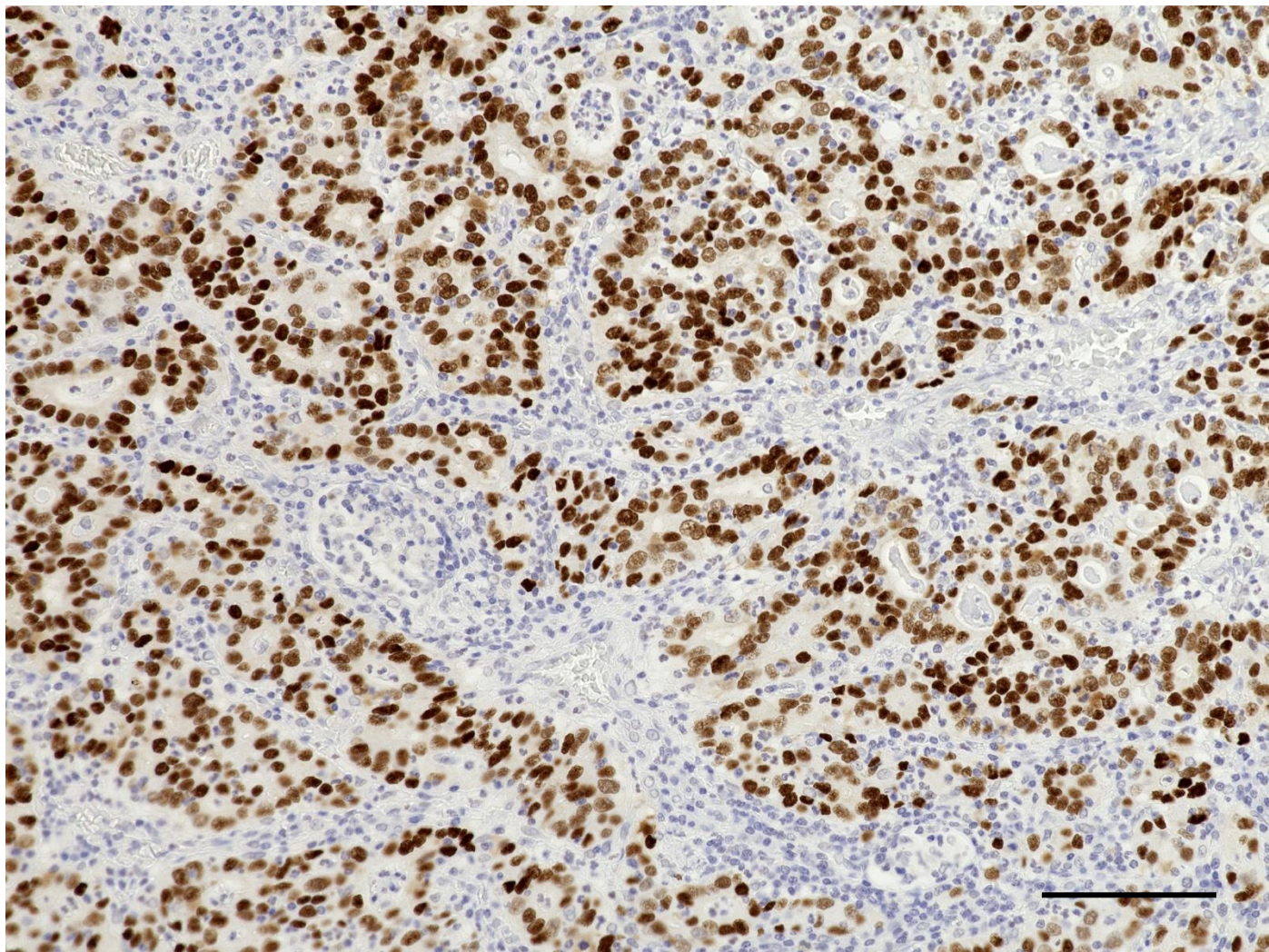

B

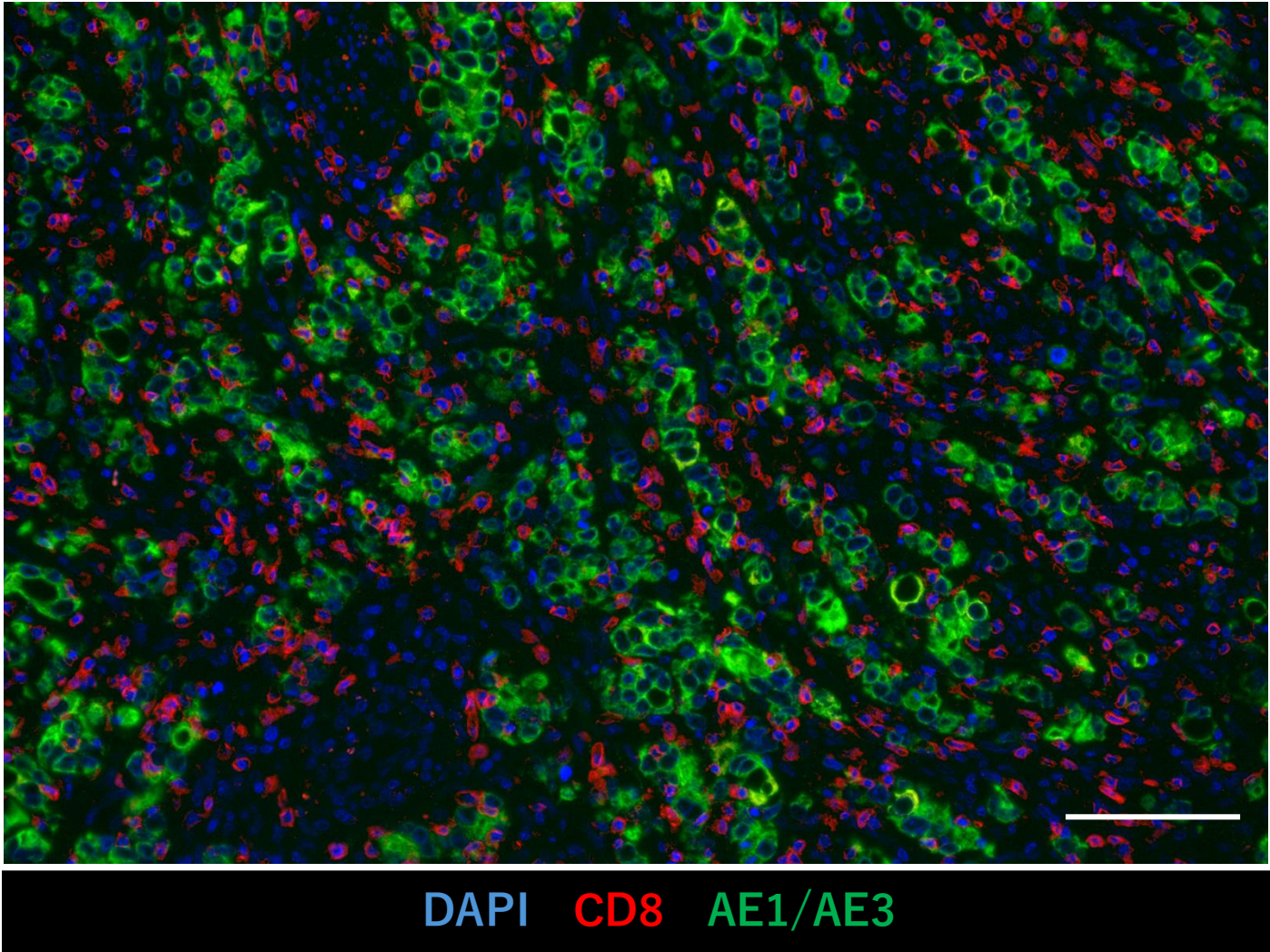

C

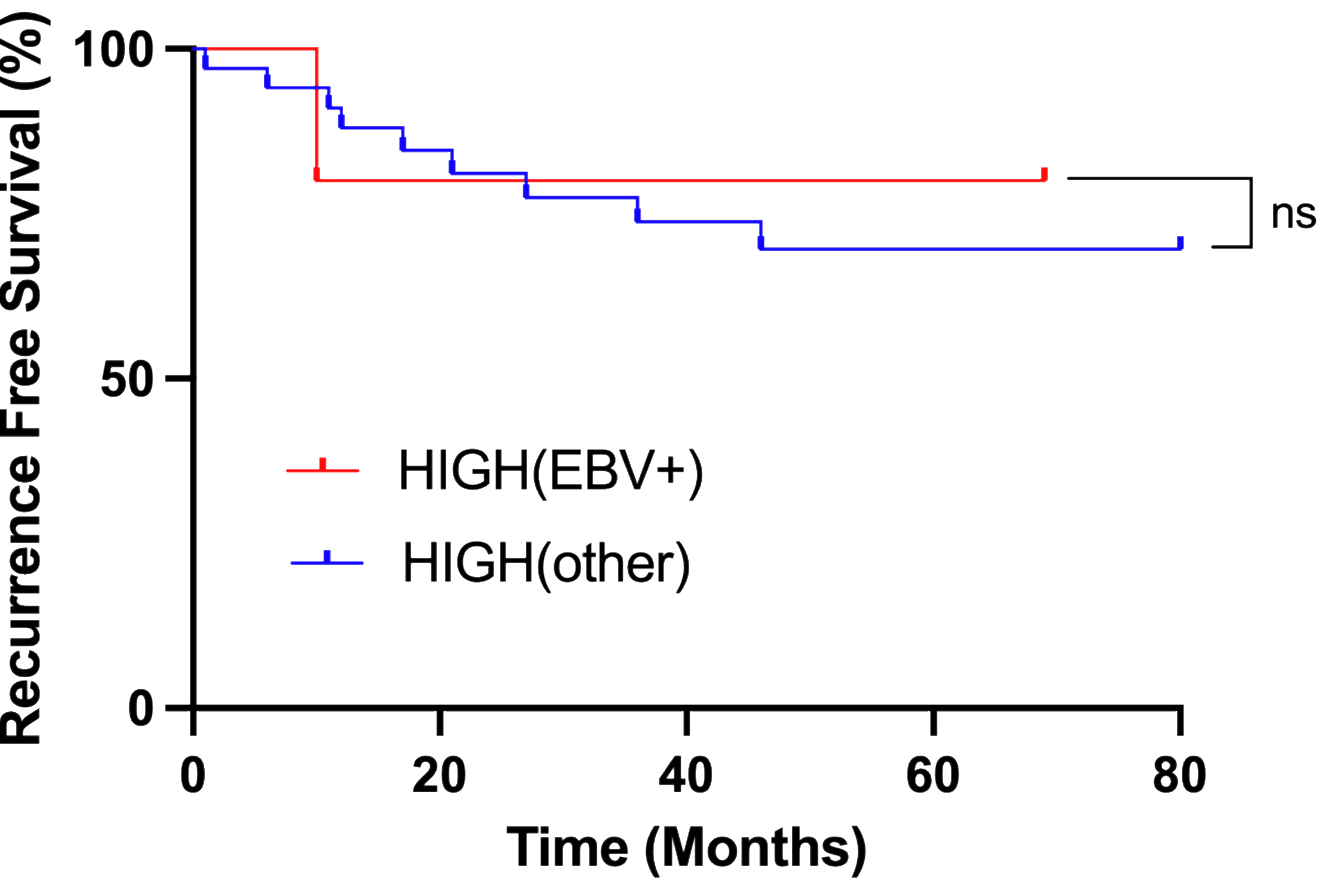

D

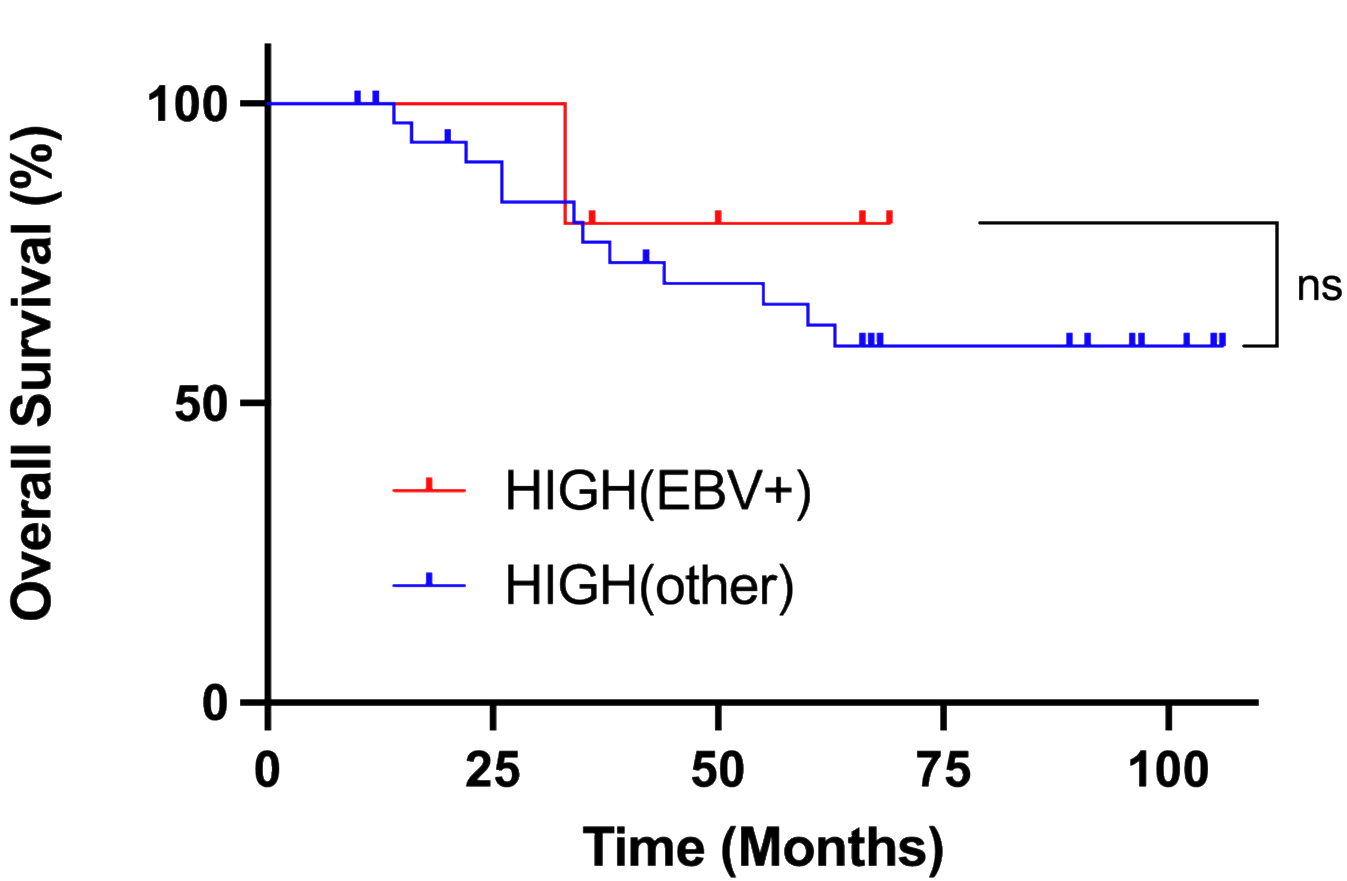

Supplement: Supplementary file 6 — Supplementary file6 (PDF 882 KB) Figure S1. Prognostic impact of the degree of CD8+ T-cell infiltration. A. Representative in situ hybridization image of Epstein–Barr-encoded RNA immunostaining of GC tissue from the HIGH (EBV) group. Scale bar, 100 μm. B. Representative multiplex immunofluorescence images of CD8+ TILs and epithelial cells using antibodies against CD8 (red) and AE1/AE3 (green), and DAPI (light blue), in GC and adjacent tissues from the HIGH (EBV) group. Scale bar, 100 μm. C. Recurrence-free survival analysis (performed using the Kaplan–Meier plotter) of patients with GC (n=39; HIGH (other) group=34, HIGH (EBV+) group=5). Statistical significance was set at p<0.05 (ns: p≥0.05, ∗: p<0.05, ∗∗: p<0.01, ∗∗∗: p<0.001, and ∗∗∗∗: p<0.0001). D. Overall survival analysis (performed using the Kaplan–Meier plotter) of patients (n=39; HIGH (other) group=34, HIGH (EBV+) group=5). Statistical significance was set at p<0.05 (ns: p≥0.05, ∗: p<0.05, ∗∗: p<0.01, ∗∗∗: p<0.001, and ∗∗∗∗: p<0.0001) [file 10120_2024_1577_MOESM6_ESM.pdf]

Figure S2

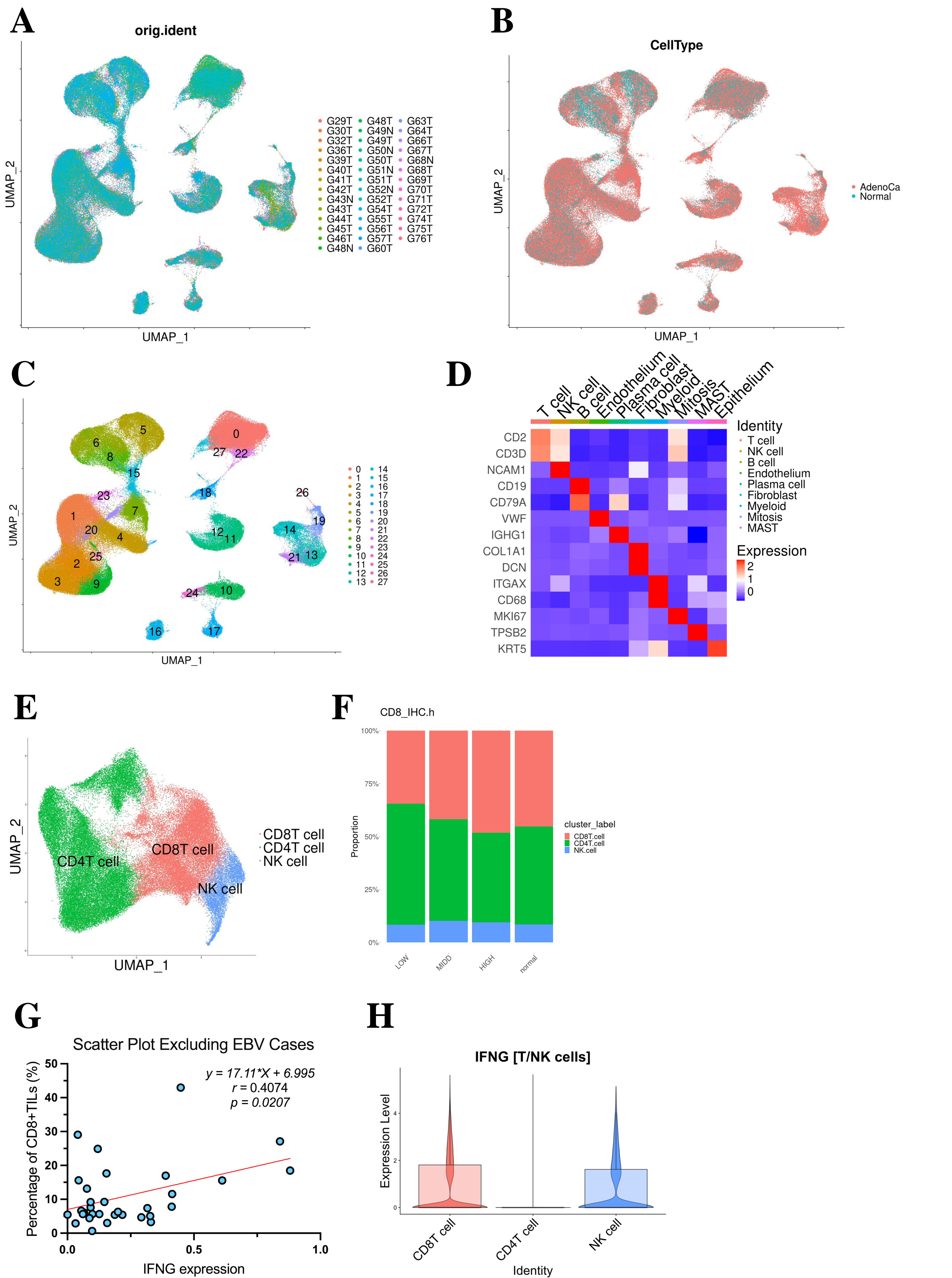

Supplement: Supplementary file 7 — Supplementary file7 (PDF 1077 KB) Figure S2. Variations in immune cell composition and IFNG expression within the GC tumor immune microenvironment. A. UMAP plot showing the clustering of cells among all cells from 41 original samples. B. UMAP plot showing the clustering of cells by the tissue source type among all cells. C. UMAP plot showing 28 clusters among all cells from 34 resected tumor tissues and 7 normal tissues. D. Heatmap of the canonical marker genes in each major cell type in all cells. E. UMAP plot of T- cells and NK cells showing clustering of CD8+ T-cells (red), CD4+ T-cells (green), and NK cells (blue). The clustering was performed to visualize the distinct populations based on their gene expression profiles. F. Bar plots showing the proportions of CD8+ T-cells, CD4+ T-cells, and NK cells across the four groups (HIGH, MID, LOW, and normal). G. Scatter plot of CD8+ T-cell percentage and IFNG expression in patients with GC excluding EBV-positive cases. The regression equation and p value are displayed in the upper right corner. H. Violin plots showing IFNG expression in CD8+ T-cells, CD4+ T-cells, and NK cells [file 10120_2024_1577_MOESM7_ESM.pdf]

Figure S3

A

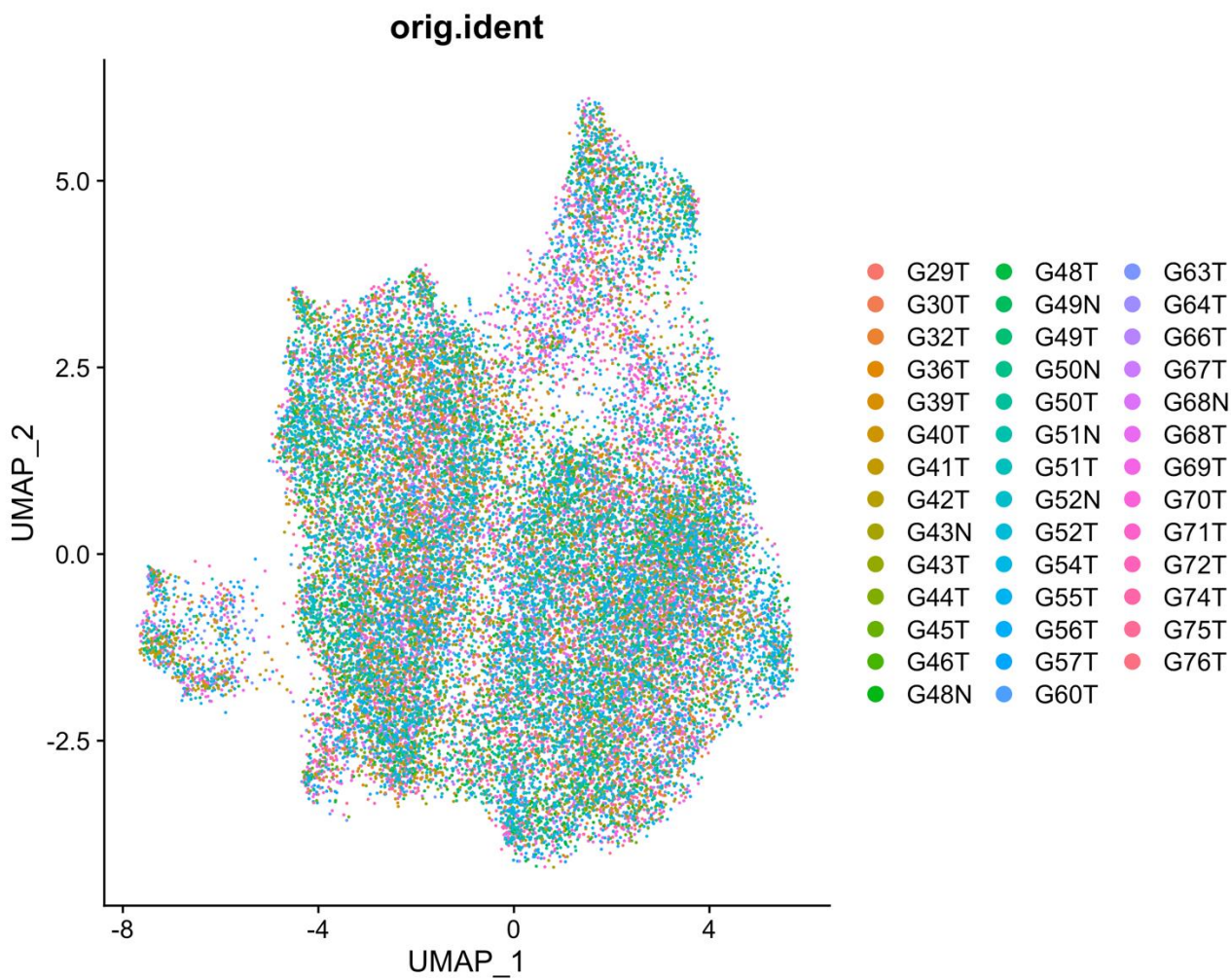

B

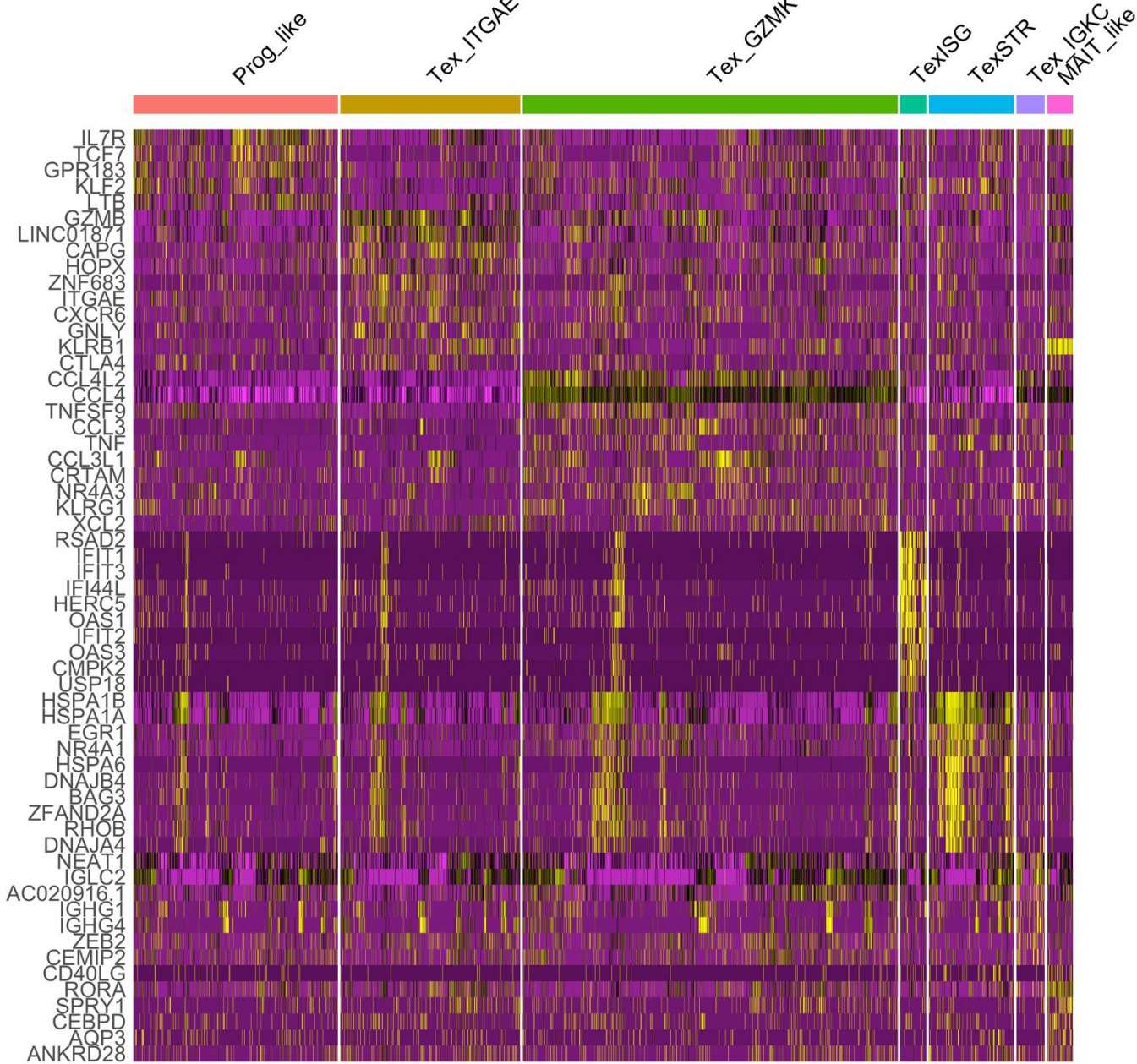

C

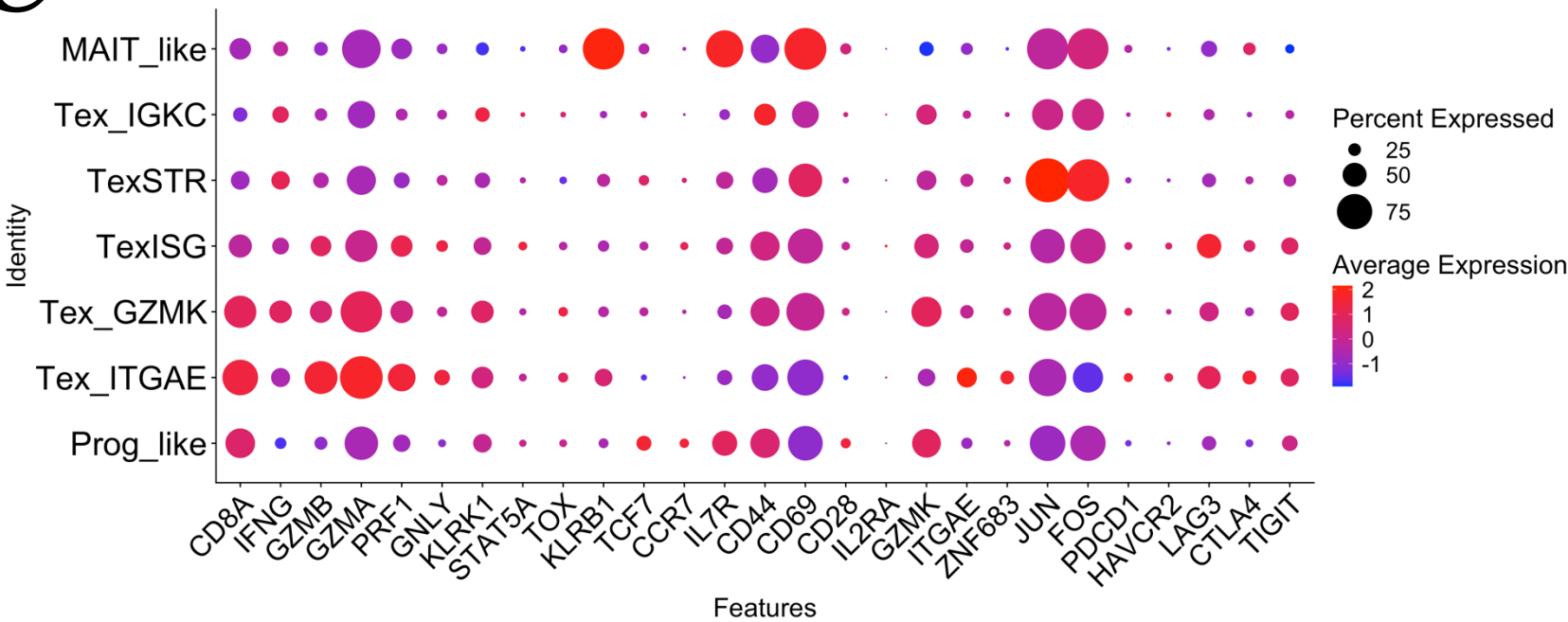

D

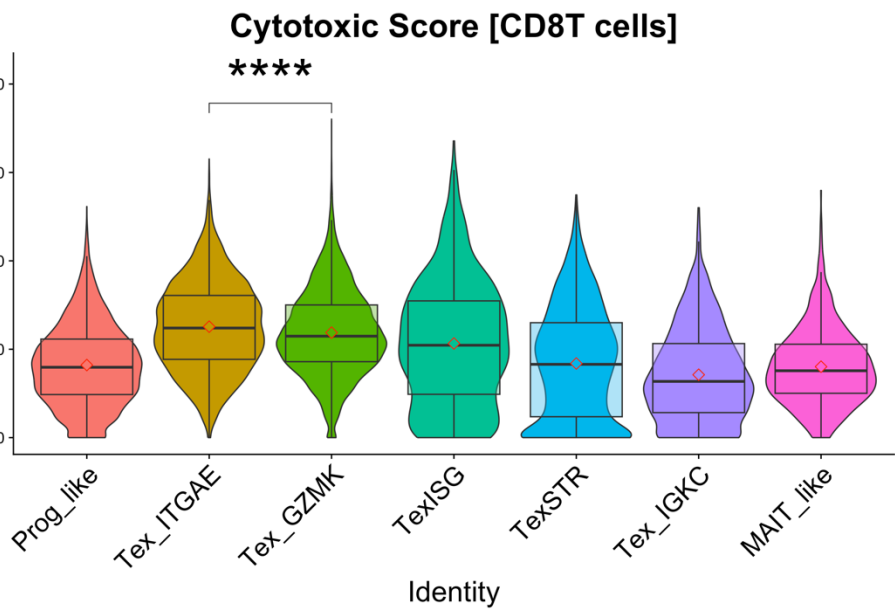

E

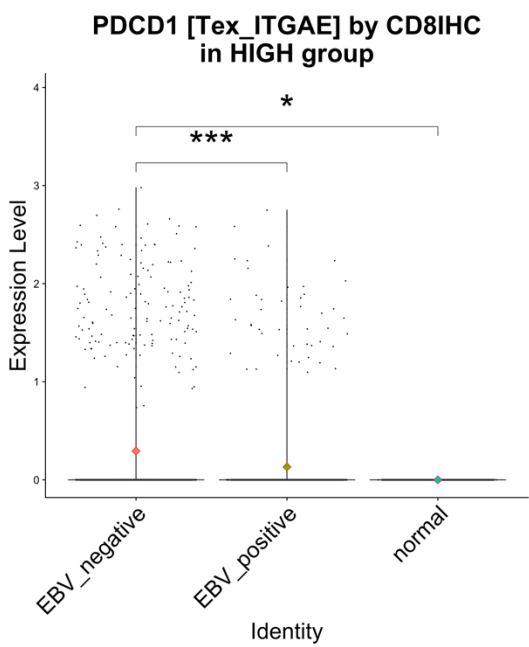

F

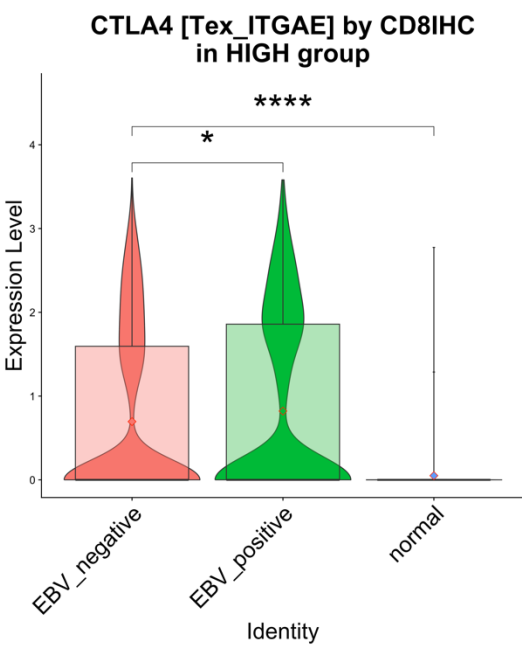

G

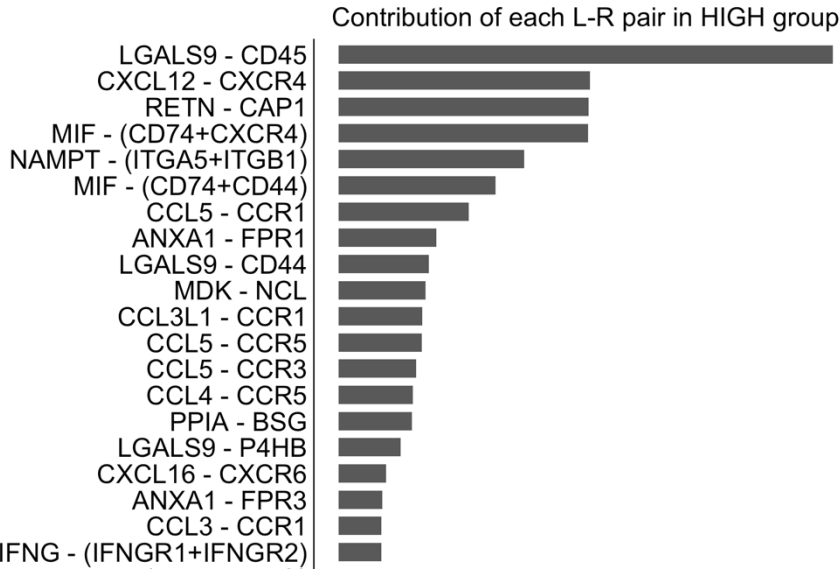

H

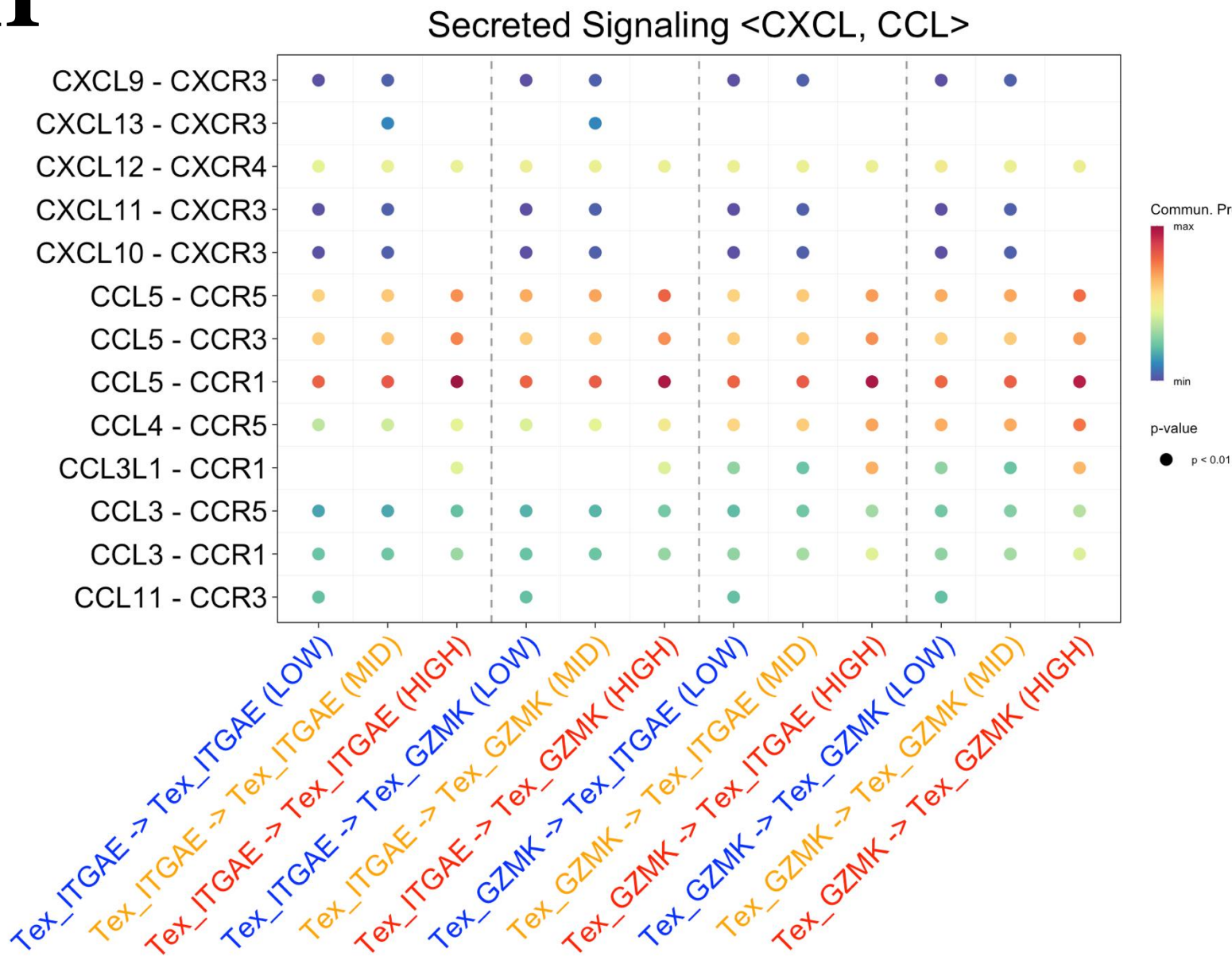

I

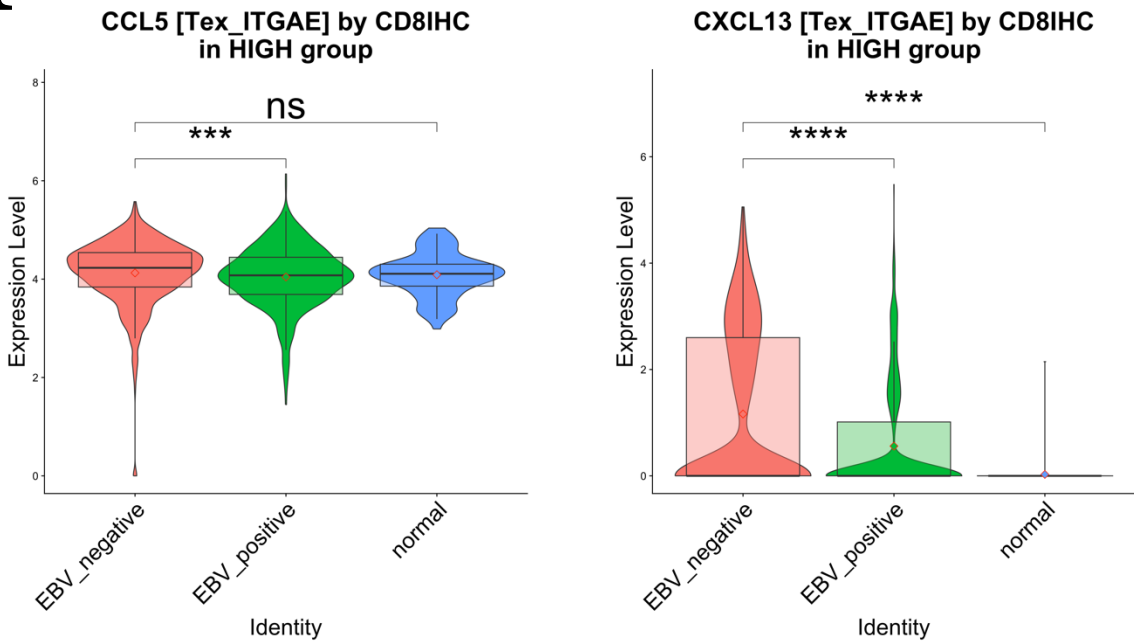

J

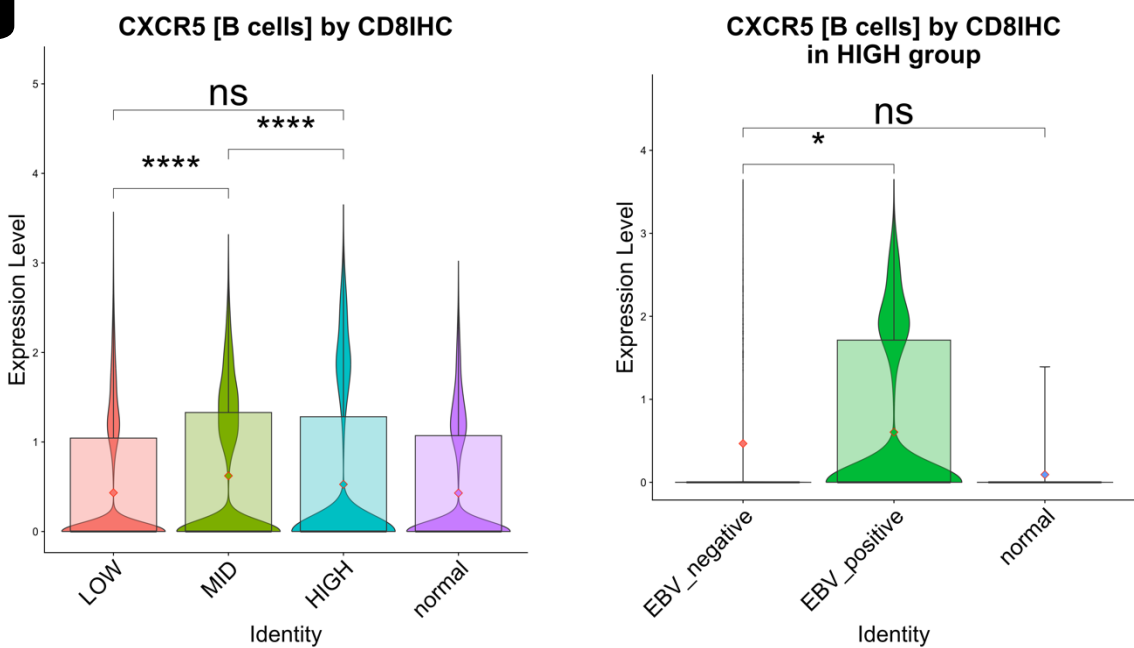

Supplement: Supplementary file 8 — Supplementary file8 (PDF 1753 KB) Figure S3. Heterogeneity in the abundance and functionality of CD8+ TILs. A. UMAP plot showing the clustering of cells among CD8+ T-cells from 41 original samples. B. Heatmap of the top 10 genes among the differentially expressed genes (DEGs) in each subtype of CD8+ T-cells. C. Dot plots showing canonical marker gene expression in the CD8+ T-cell subtypes. D. Violin plots showing the cytotoxic scores of the CD8+ T-cell subtypes. Statistical significance was set at p<0.05 (ns: p≥0.05, ∗: p<0.05, ∗∗: p<0.01, ∗∗∗: p<0.001, and ∗∗∗∗: p<0.0001). E. Violin plots showing PDCD1 (left) and CTLA4 (right) expression in the Tex_ITGAE subtype across three HIGH groups (EBV-positive, EBV-negative, and normal). Individual points represent single cells. Statistical significance was set at p<0.05 (ns: p≥0.05, ∗: p<0.05, ∗∗: p<0.01, ∗∗∗: p<0.001, and ∗∗∗∗: p<0.0001). F. Violin plots showing TIGIT (left) and LAG3 (right) expression in the Tex_ITGAE subtype across four groups (HIGH, MID, LOW, and normal). Statistical significance was set at p<0.05 (ns: p≥0.05, ∗: p<0.05, ∗∗: p<0.01, ∗∗∗: p<0.001, and ∗∗∗∗: p<0.0001). G. Ranking of the top 10 ligand–receptor pairs in all cell clusters within the HIGH group using CellChat. H. Dot plots showing the secreted signaling pathways (CXCL and CCL) in the Tex_ITGAE and Tex_GZMK subtypes across three groups (HIGH, MID, and LOW) using CellChat. I. Violin plots showing CCL5 (left) and CXCL13 (right) expression in the Tex_ITGAE subtype across three HIGH groups (EBV-positive, EBV-negative, and normal). Statistical significance was set at p<0.05 (ns: p≥0.05, ∗: p<0.05, ∗∗: p<0.01, ∗∗∗: p<0.001, and ∗∗∗∗: p<0.0001). J. Violin plots showing CXCR5 expression by B-cells across four groups (HIGH, MID, LOW, and normal; left) and three HIGH groups (EBV-positive, EBV-negative, and normal; right). Statistical significance was set at p<0.05 (ns: p≥0.05, ∗: p<0.05, ∗∗: p<0.01, ∗∗∗: p<0.001, and ∗∗∗∗: p<0.0001) [file 10120_2024_1577_MOESM8_ESM.pdf]

# Figure S4

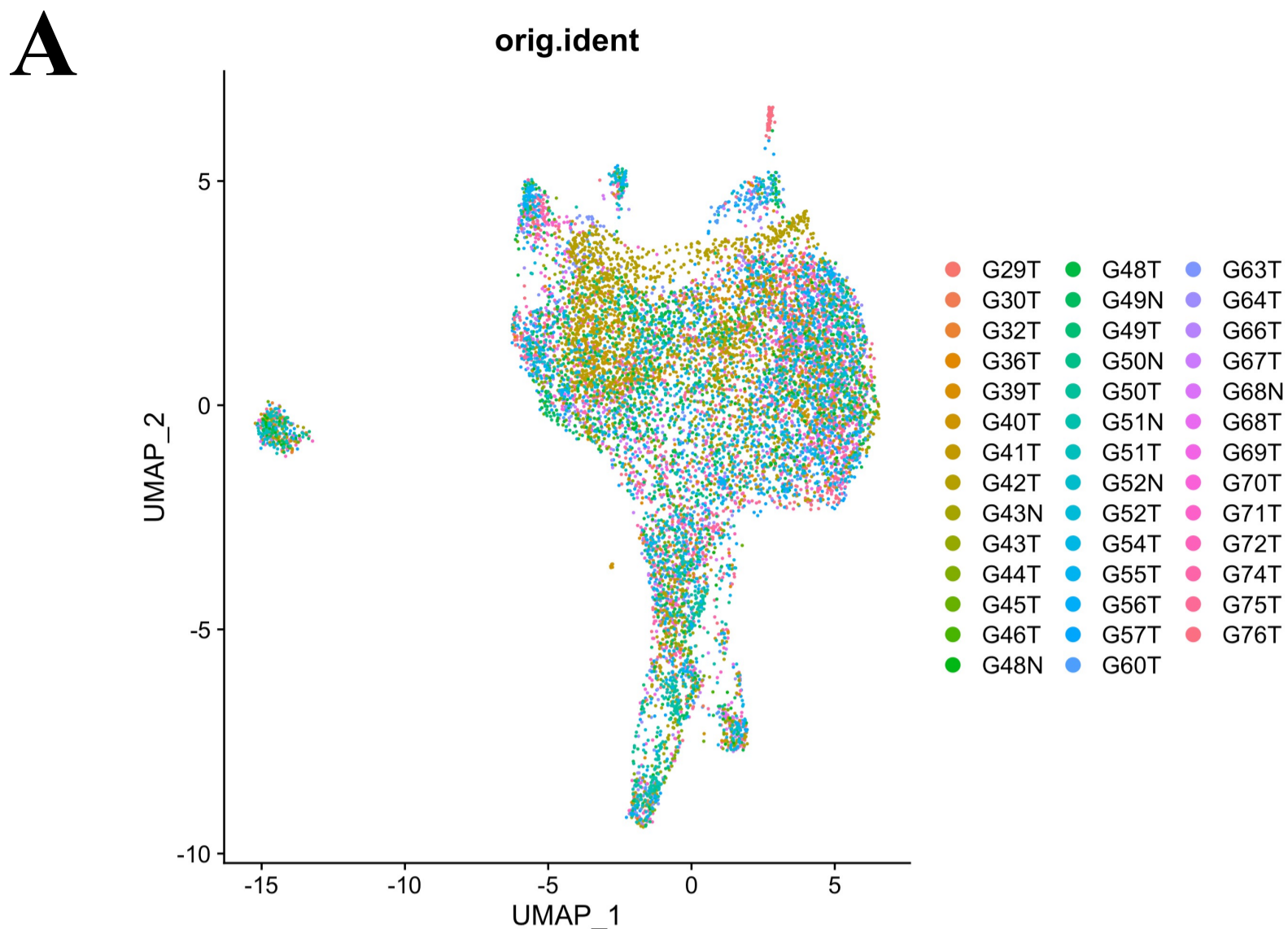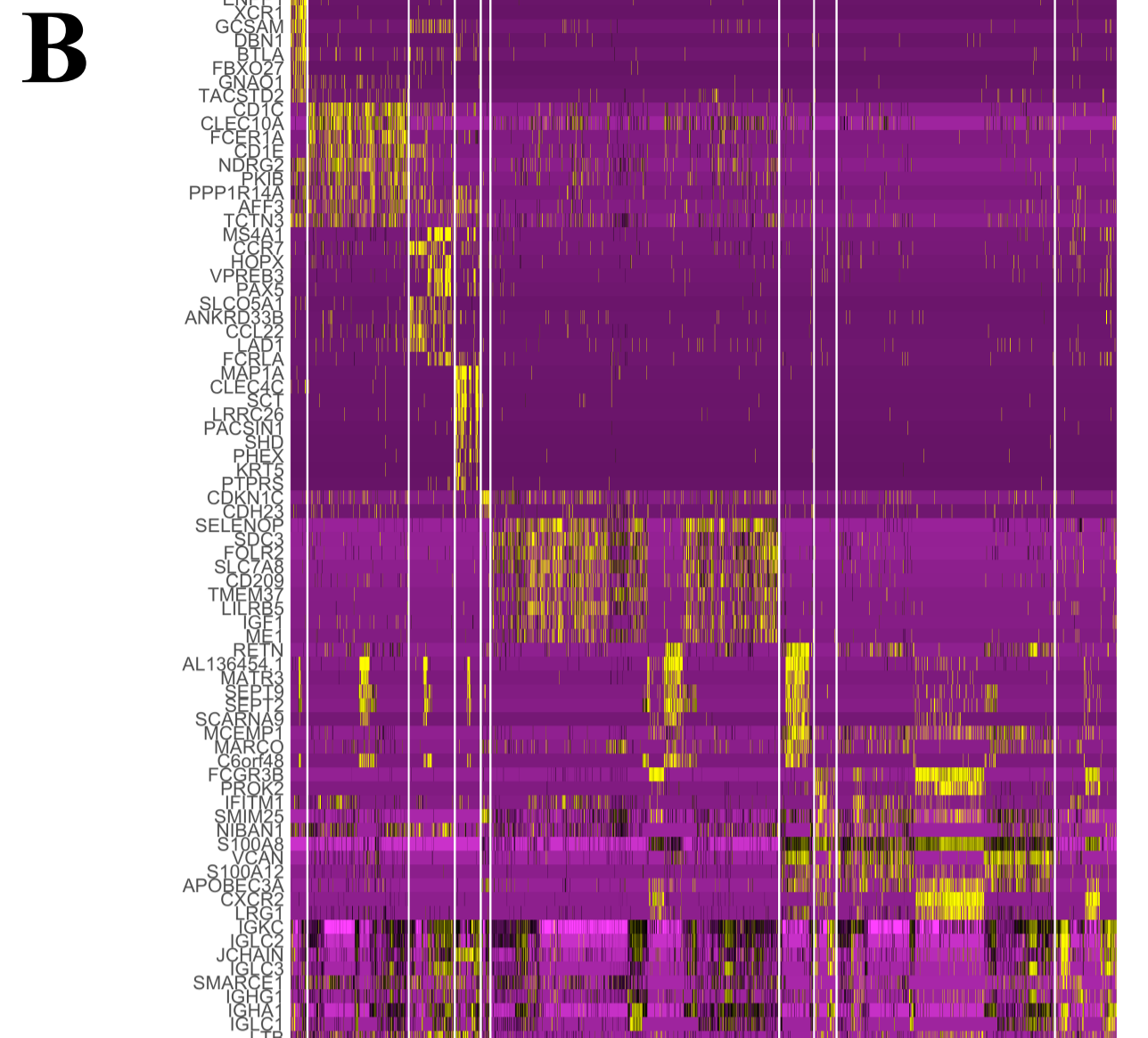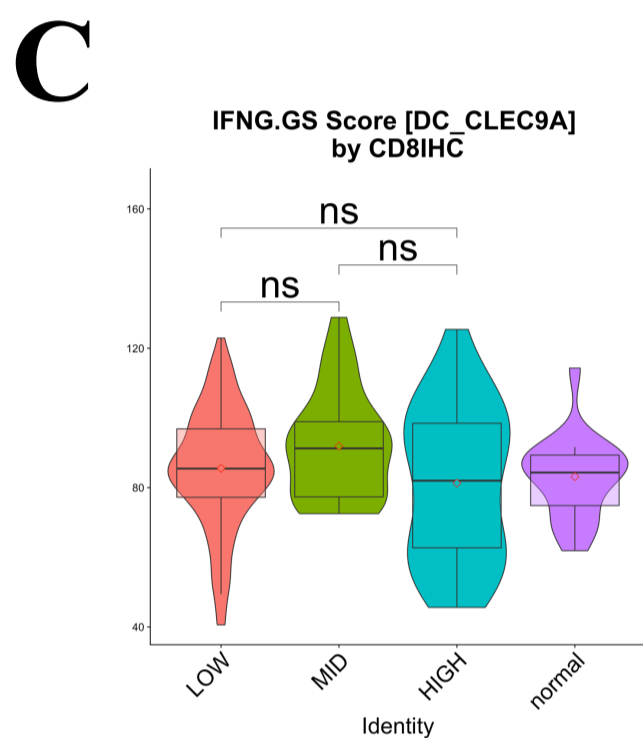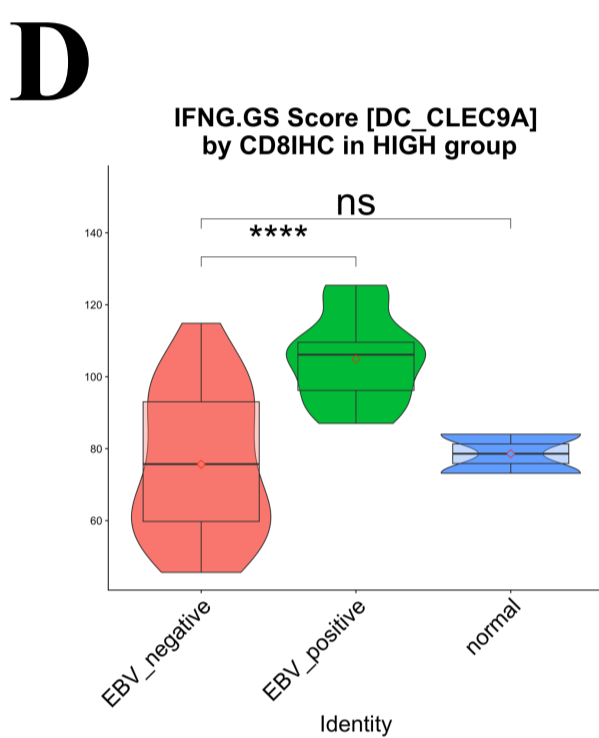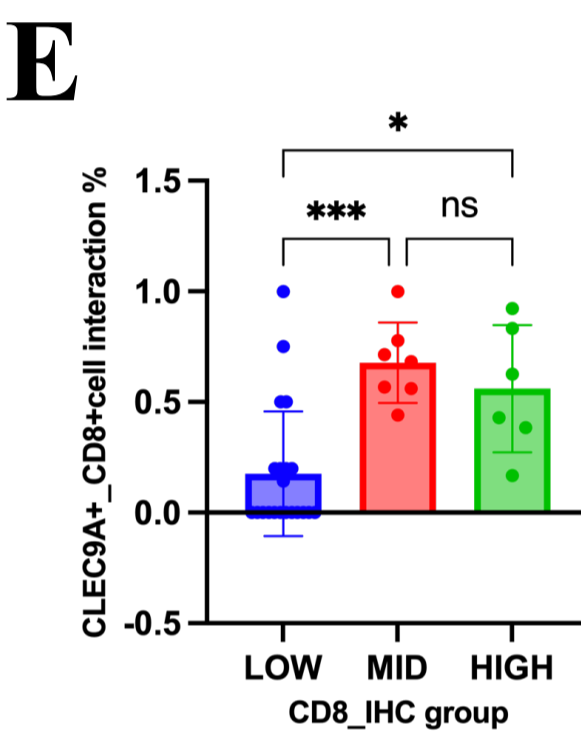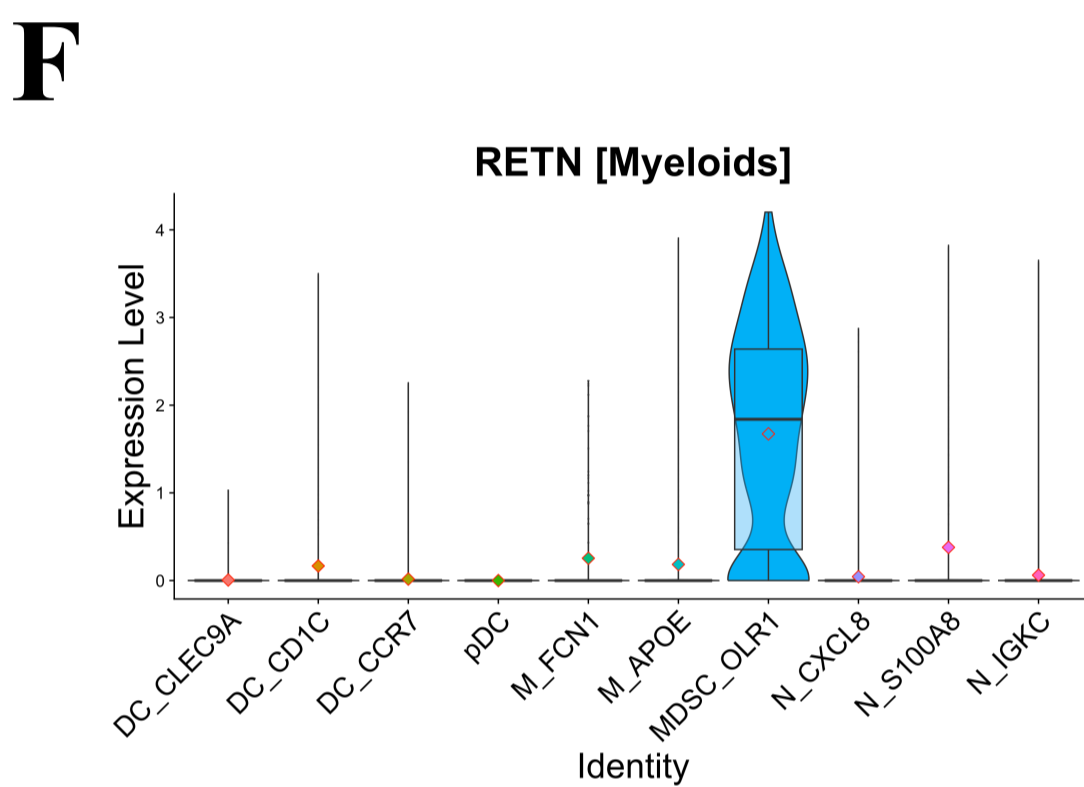

Supplement: Supplementary file 9 — Supplementary file9 (PDF 2381 KB) Figure S4. Characterization of the dynamics and pathway interactions of myeloid cells. A. UMAP plot showing the clustering of cells among myeloid cells from 41 original samples. B. Heatmap of the top 10 genes among the DEGs in each subtype of myeloid cells. C. Violin plots showing the IFNG.GS scores of the DC_CLEC9A subtype across four groups (HIGH, MID, LOW, and normal). Statistical significance was set at p<0.05 (ns: p≥0.05, ∗: p<0.05, ∗∗: p<0.01, ∗∗∗: p<0.001, and ∗∗∗∗: p<0.0001). D. Violin plots showing the IFNG.GS scores of the DC_CLEC9A subtype across three HIGH groups (EBV-positive, EBV-negative, and normal). Statistical significance was set at p<0.05 (ns: p≥0.05, ∗: p<0.05, ∗∗: p<0.01, ∗∗∗: p<0.001, and ∗∗∗∗: p<0.0001). E. Quantification bar plot of interaction counts between CLEC9A+ cells and CD8+ cells across three groups (HIGH, MID, and LOW). The interaction counts were defined as the presence of one or more CD8+ cells within a 20-μm radius centered on the CLEC9A+ cells. Statistical significance was set at p<0.05 (ns: p≥0.05, ∗: p<0.05, ∗∗: p<0.01, ∗∗∗: p<0.001, and ∗∗∗∗: p<0.0001). F. Violin plots showing RETN expression in the myeloid cell clusters [file 10120_2024_1577_MOESM9_ESM.pdf]

Figure S5

A

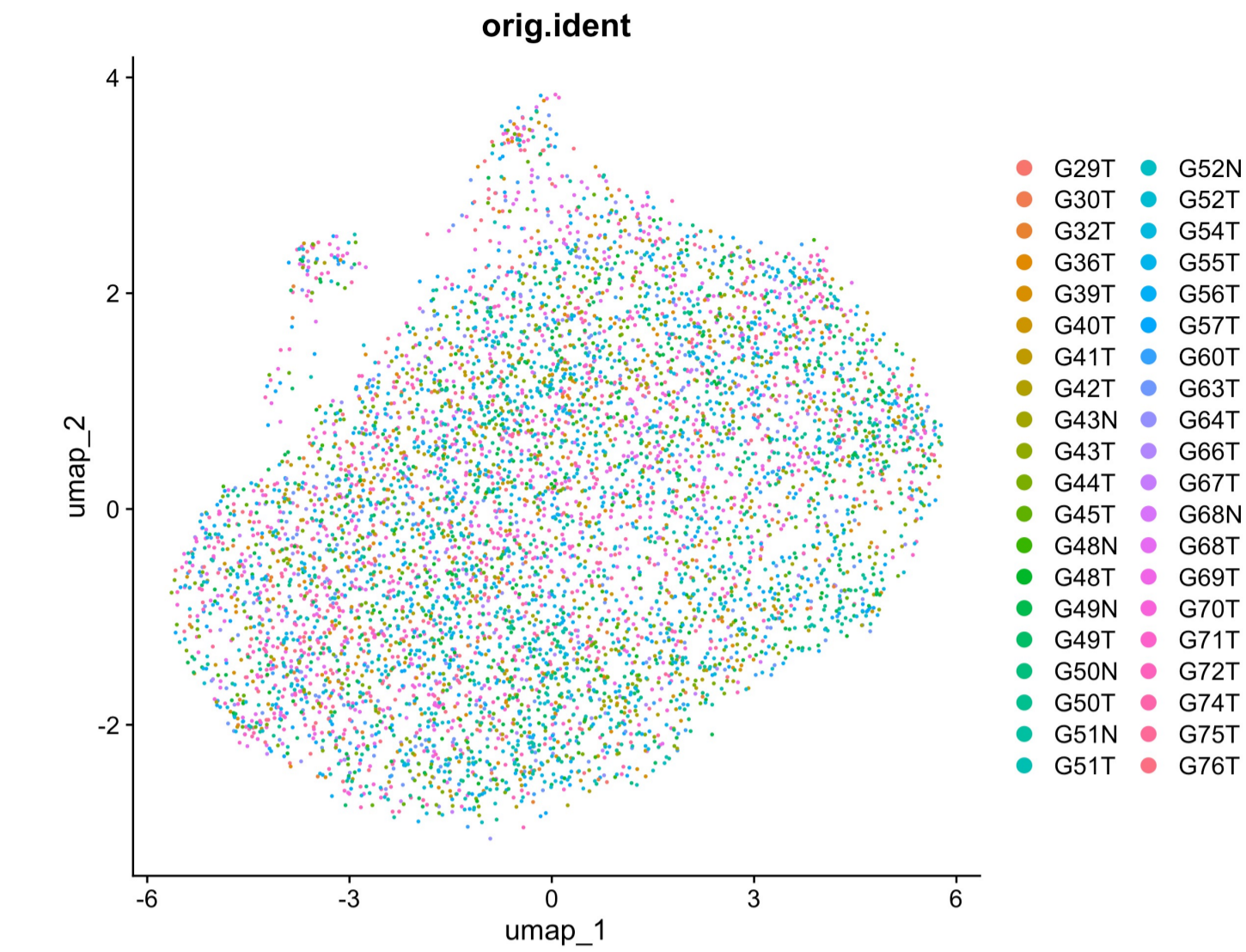

B

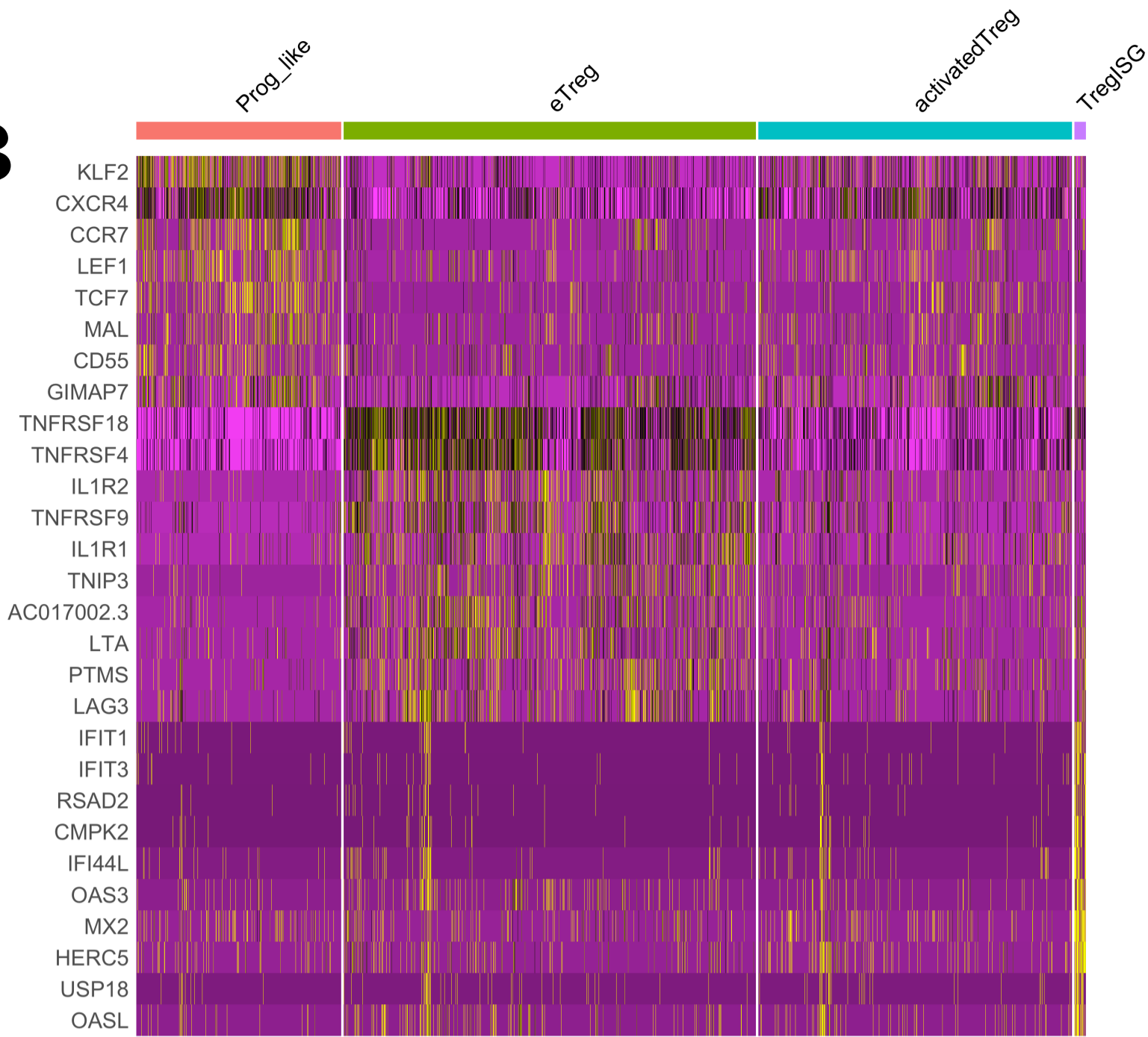

C

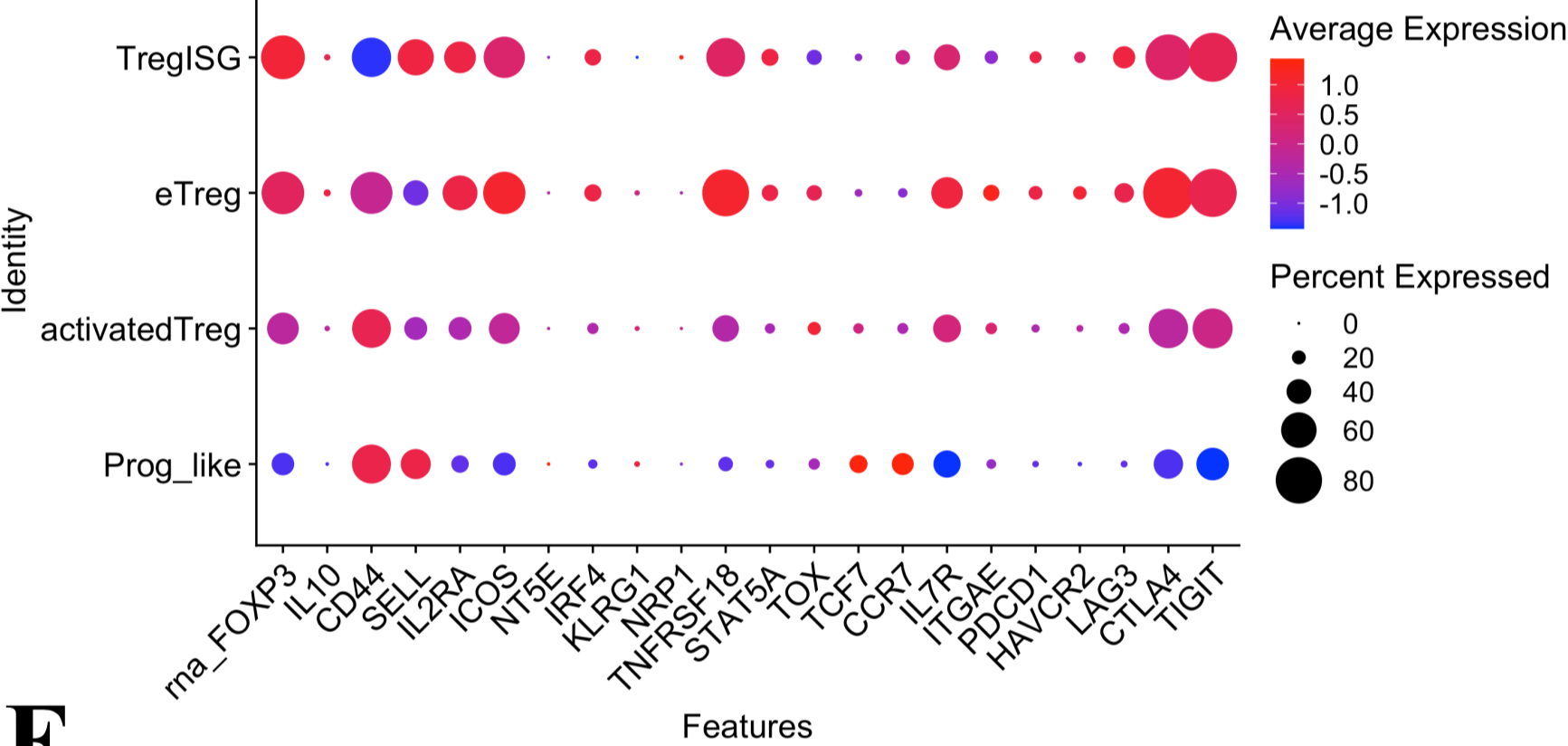

E

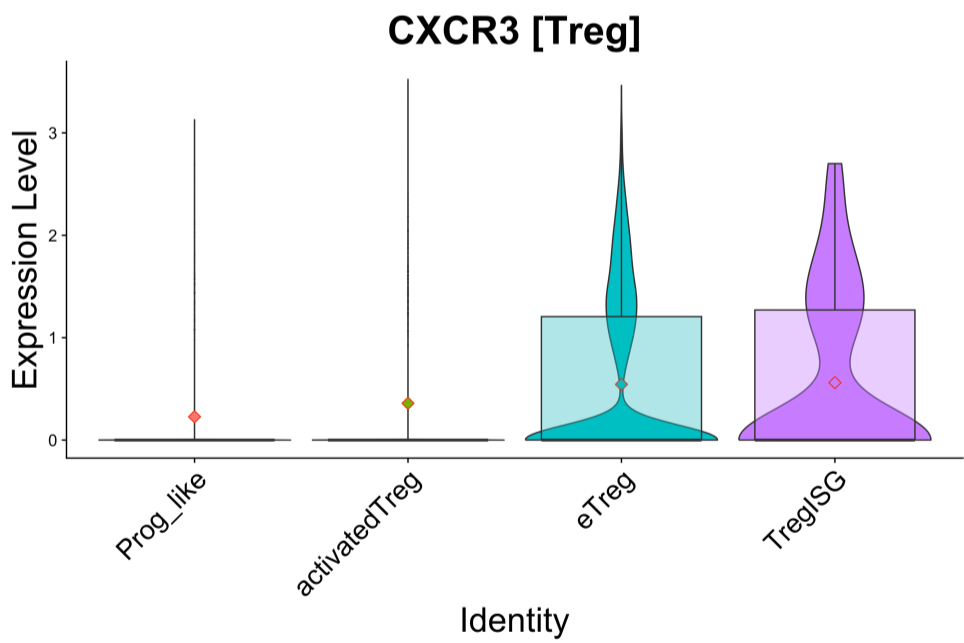

D

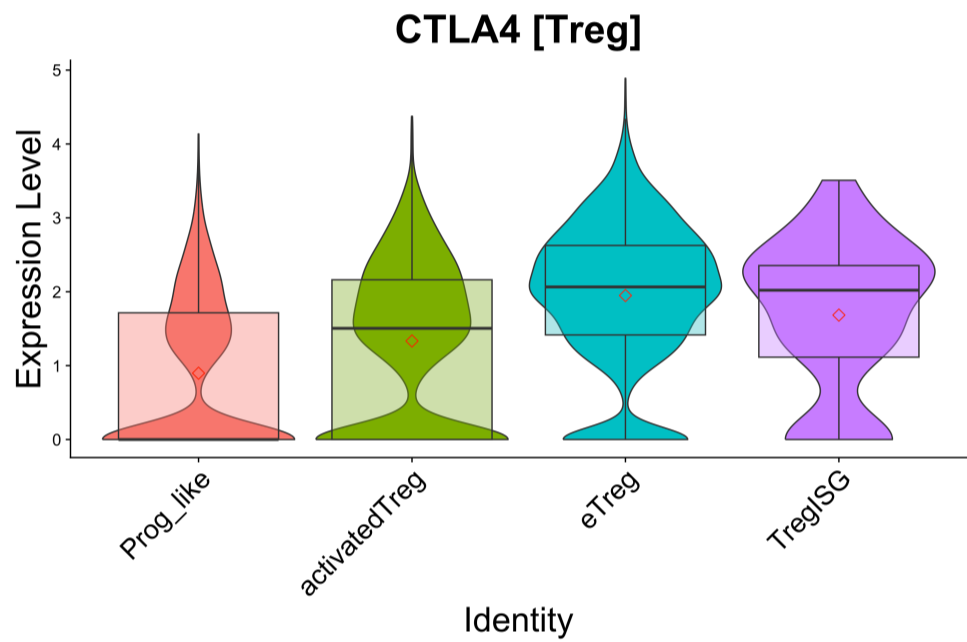

Supplement: Supplementary file 10 — Supplementary file10 (PDF 2483 KB) Figure S5. Analysis of Treg subclusters and CTLA4 expression across different CD8+ TIL groups. A. UMAP plot showing the clustering of cells among Tregs from 41 original samples. B. Heatmap of the top 10 genes from the DEGs in each subtype in Tregs. C. Dot plots showing canonical marker gene expression in the Treg subtypes. D. Violin plots showing CTLA4 expression in the Treg subtypes. E. Violin plots showing CXCR3 expression in the Treg subtypes [file 10120_2024_1577_MOESM10_ESM.pdf]

Figure S6

A

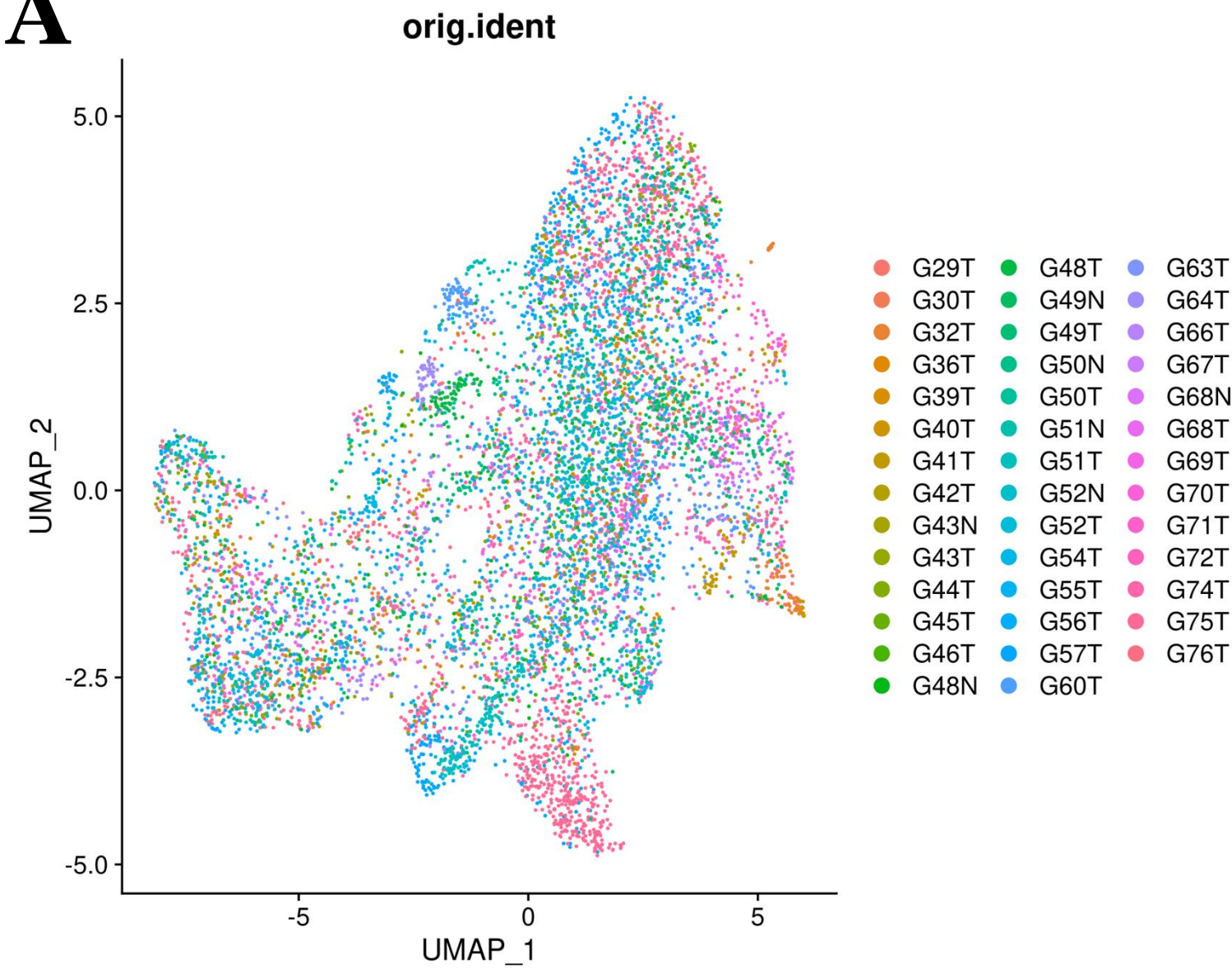

B

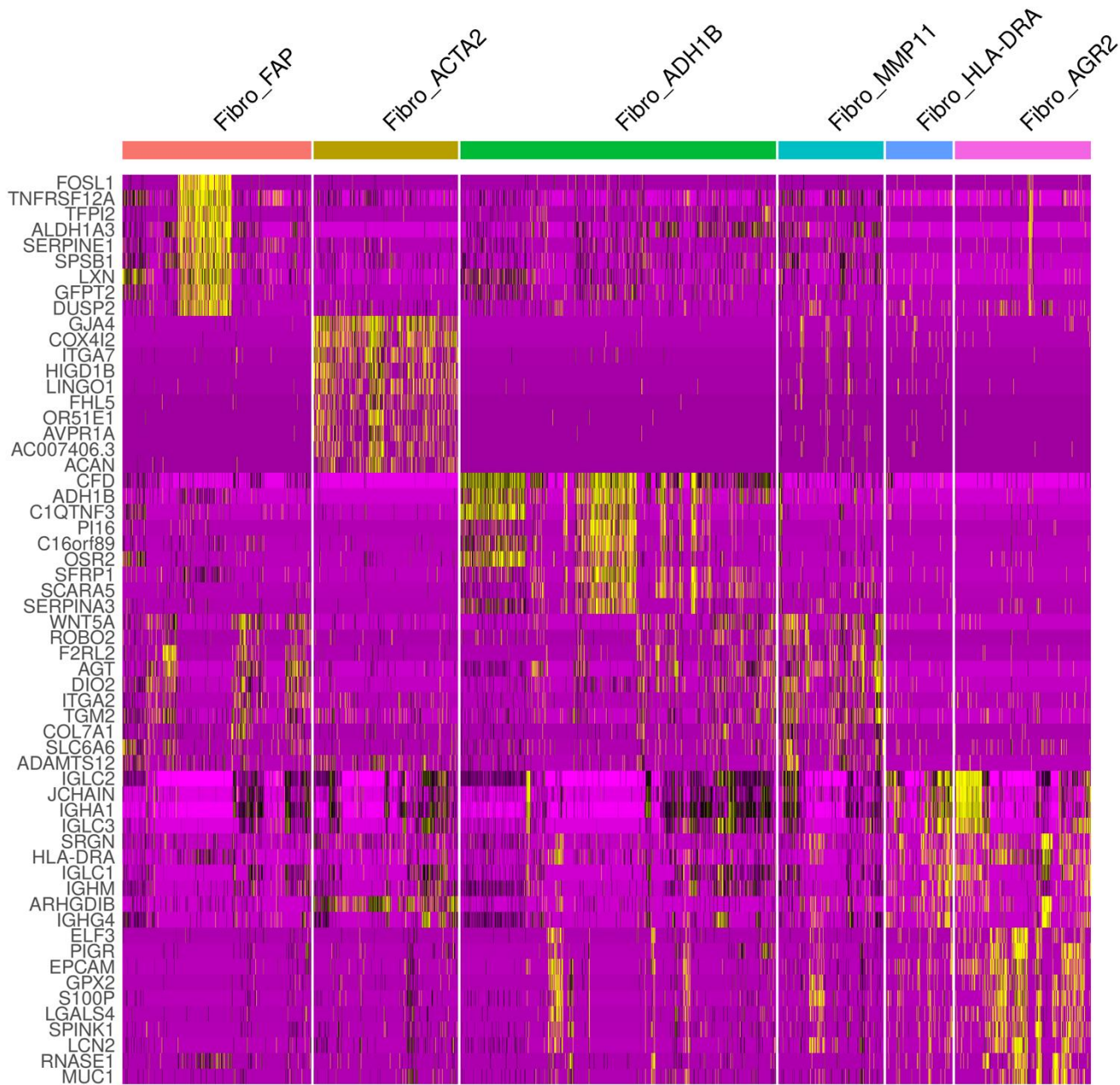

C

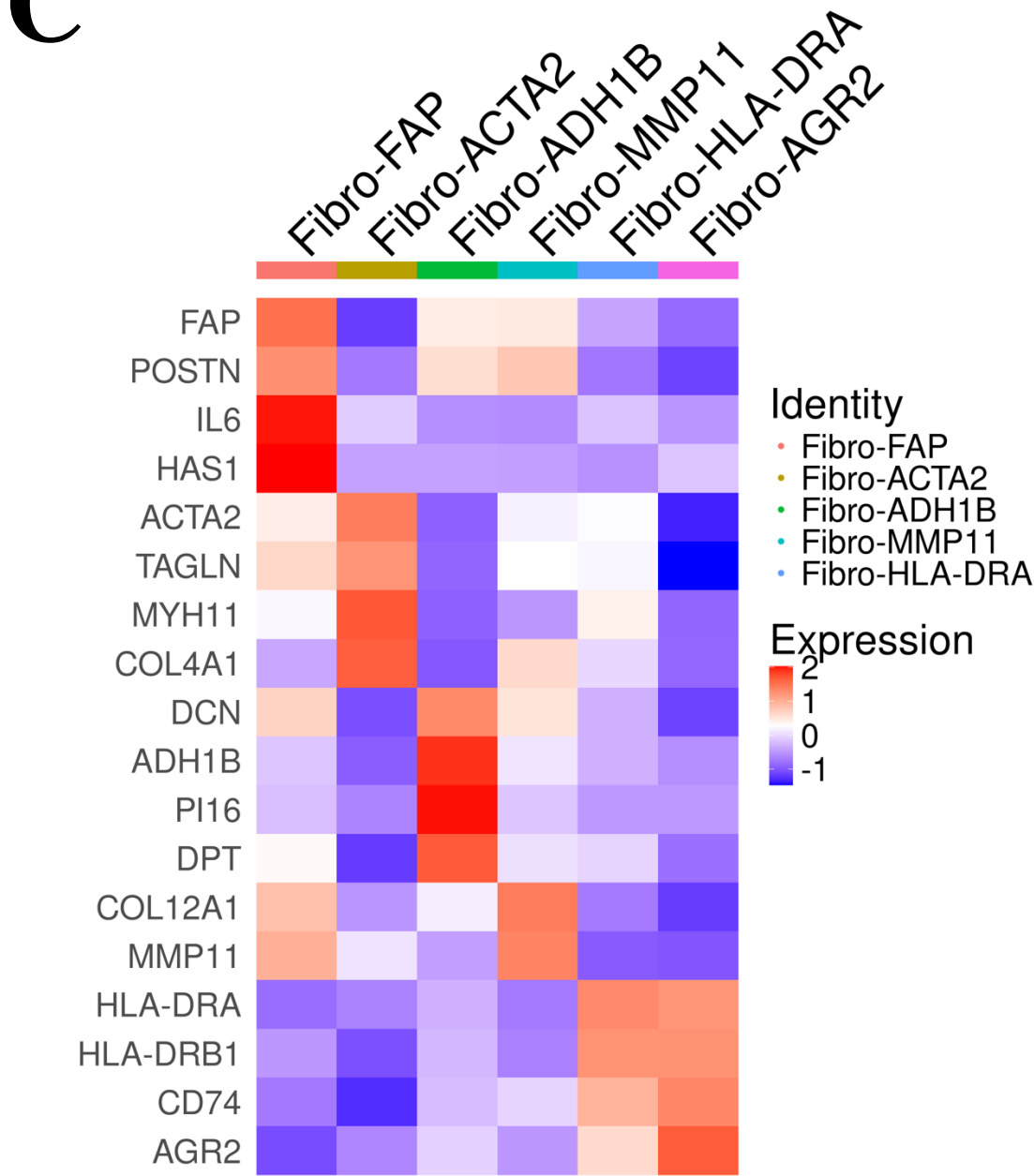

Supplement: Supplementary file 11 — Supplementary file11 (PDF 798 KB) Figure S6. Immunosuppressive roles of fibroblasts in the GC microenvironment. A. UMAP plot showing the clustering of cells among fibroblasts from 41 original samples. B. Heatmap of the top 10 genes from the DEGs in each subtype in fibroblasts. C. Heatmap of the canonical marker genes in each subtype in fibroblasts [file 10120_2024_1577_MOESM11_ESM.pdf]
